# Efficacy of acupuncture therapies for polycystic ovary syndrome: a network meta-analysis

**DOI:** 10.3389/fmed.2026.1895421

**Published:** 2026-07-29

**Authors:** Ning Yang, Yunxi Li, Hailin Jiang, Rulin Wang, Qi Zhang, Qingqing Tang, Huiying Liu, Yuhong Xie, Sixian Wang, Zhijie Li, Zhihao Dong, Jinying Zhao, Fuchun Wang

**Affiliations:** 1 Acupuncture and Massage College, Changchun University of Chinese Medicine, Changchun, Jilin, China; 2 Shenzhen Traditional Chinese Medicine Hospital, Shenzhen, Guangdong, China

**Keywords:** acupuncture-related therapy, network meta-analysis, polycystic ovary syndrome, sex hormones, total efficacy rate

## Abstract

**Background:**

Polycystic ovary syndrome (PCOS) is a prevalent gynecological disorder that significantly impairs quality of life and reduces patient confidence in treatment. The efficacy of current Western pharmacological therapies is limited. Acupuncture has shown promise in the management of PCOS, but comparative research on different acupuncture modalities remains scarce.

**Methods:**

PubMed, EMBASE, Cochrane Library, Web of Science, CNKI, Wanfang, VIP, and the Chinese Biomedical Literature Database were comprehensively searched from database inception to 18 August 2024. Two reviewers independently screened studies, extracted data, and assessed the risk of bias in the eligible studies. Network meta-analysis was conducted using STATA 15.1 and R 4.4.1.

**Results:**

Ninety-two randomized controlled trials (RCTs) involving 15 acupuncture-based interventions or combinations were included. According to the surface under the cumulative ranking curve (SUCRA) analysis, for total efficacy (TE), electroacupuncture (99.03%), filiform needle + thunder fire moxibustion (74.77%), and filiform needle with electroacupuncture (64.02%) ranked the highest. For follicle-stimulating hormone (FSH) regulation, fire needle (90.99%) ranked first, followed by filiform needle + electroacupuncture (75.29%) and filiform needle + warming needle moxibustion + electroacupuncture (66.76%). For luteinizing hormone (LH) regulation, warming needle moxibustion (86.75%) ranked first, followed by warming needle moxibustion + electroacupuncture (72.76%) and filiform needle alone (69.55%). For testosterone reduction, filiform needle + moxibustion (96.53%) ranked first, followed by catgut embedment in acupoint + moxibustion (85.64%) and electroacupuncture (82.33%). For LH/FSH ratio improvement, catgut embedment in acupoint (77.73%), warming needle moxibustion + electroacupuncture (72.87%), and filiform needle + warming needle moxibustion + electroacupuncture (66.39%) ranked the highest. For adverse events, fire needle (91.69%) ranked highest, followed by Western medicine (83.17%) and catgut embedment in acupoint (64.08%).

**Conclusion:**

No statistically significant differences were observed between single and combined acupuncture therapies. However, modalities incorporating electrical, thermal, or sustained stimulation appear to be more effective. Given the methodological limitations of the included studies, high-quality, rigorously designed RCTs are warranted to validate these findings.

**Systematic review registration:**

PROSPERO, CRD42024609903.

## 1 Background

Polycystic ovary syndrome (PCOS) is the most common endocrine disorder in women of reproductive age, with a global prevalence estimated at 5–18% (1). Up to 70% of affected individuals remain undiagnosed (2). PCOS is characterized by hormonal imbalance, chronic anovulation, and polycystic ovarian morphology, resulting in substantial physical and psychological burden (3). Its etiology is complex and not yet fully understood, and the condition is highly recurrent and often resistant to standard therapies (4). Hyperandrogenemia-induced hormonal dysregulation is considered a key pathogenic mechanism (5).

Current management primarily relies on pharmacological therapy, often combined with lifestyle modification, including exercise and dietary interventions. Commonly used drugs include ovulation-inducing agents, selective estrogen receptor modulators, and biguanides (6). However, these approaches are frequently limited in efficacy and may cause adverse effects such as chest discomfort and gastrointestinal disturbances (7). Accordingly, exploring safe and effective alternative treatment strategies is urgent. Acupuncture addresses internal disorders by external treatment methods, for instance, using specific needling techniques to externally stimulate meridians and acupoints (8). Studies (9, 10) suggest that acupuncture can improve outcomes in PCOS, particularly by modulating the sympathetic nervous system and endocrine function to regulate hormone secretion and promote ovulation, with sustained benefits and few adverse effects.

Several systematic reviews and pairwise meta-analyses have evaluated acupuncture for PCOS in recent years. However, most of these studies assessed only a limited range of acupuncture modalities and were restricted to direct comparisons, usually between one acupuncture-related therapy and Western medicine. Given the diversity of acupuncture techniques, as well as the scarcity of direct head-to-head trials among different modalities, conventional meta-analysis approaches are insufficient to determine their relative efficacy and safety. Network meta-analysis extends pairwise meta-analysis by integrating both direct and indirect evidence across multiple interventions, thereby allowing comparative ranking of treatment options within a unified analysis framework. Nevertheless, network meta-analysis needs to meet methodological assumptions, including transitivity, consistency, and heterogeneity assumptions, because bias may propagate through indirect comparisons. Therefore, a network meta-analysis is particularly needed to comprehensively compare acupuncture-related therapies for PCOS (11–14).

To address these gaps, the present study conducted a network meta-analysis to compare multiple acupuncture therapies in terms of regulating sex hormone levels, overall clinical efficacy, and safety in PCOS patients. Furthermore, these interventions were ranked based on related data, with the aim of providing evidence-based guidance for optimizing acupuncture treatment in clinical practice.

## 2 Data and methods

### 2.1 Methods

This network meta-analysis was conducted in accordance with the Preferred Reporting Items for Systematic Reviews and Meta-Analyses (PRISMA), including the PRISMA extension for network meta-analyses (15). The study protocol was prospectively registered with the International Prospective Register of Systematic Reviews (PROSPERO) (CRD42024609903).

### 2.2 Search strategy

PubMed, Embase, Cochrane Library, Web of Science, CNKI, Wanfang Data, VIP, and the Chinese Biomedical Literature Database (CBM) were comprehensively searched from database inception to 18 August 2024 to collect related publications written in English and Chinese. Medical subject headings (MeSH) and free-text terms were combined to design search terms. MeSH terms encompassed acupuncture, acupuncture therapy, acupoint catgut embedding therapy, moxibustion, warming needle moxibustion, cystic ovary, and micropolycystic ovary. Additionally, reference lists of relevant studies and gray literature sources were manually screened to identify additional eligible studies. The full search strategy is provided in Supplementary Table 1.

### 2.3 Eligibility criteria

Studies were eligible for inclusion if they met the following criteria: (1) Participants: Women diagnosed with PCOS according to the Rotterdam criteria established by the European Society of Human Reproduction and Embryology (ESHRE) and the American Society for Reproductive Medicine (ASRM) in January 2003 (16). (2) Interventions: Control groups received ovulation-inducing pharmacotherapy. Intervention groups received one or more acupuncture-related therapies in combination with ovulation-inducing drugs, including filiform needle (FN), electroacupuncture, warming needle moxibustion (WNM), catgut embedment in acupoint (CEIA), moxibustion, fire needle, ear acupuncture (EA), or thunder fire moxibustion (TFM). (3) Study design: Randomized controlled trials (RCTs) only. (4) Outcomes: Primary outcomes encompassed follicle-stimulating hormone (FSH), luteinizing hormone (LH), testosterone, and LH/FSH ratio. Secondary outcomes included overall clinical effectiveness and adverse events. Total efficacy (TE) was assessed based on predefined criteria.

Studies were excluded if they met any of the following criteria: (1) non-clinical studies (e.g., animal or *in vitro* experiments), case reports, study protocols, reviews, letters, editorials, or conference abstracts; (2) studies with incomplete or seriously flawed data; (3) duplicate publications; (4) full text unavailable; or (5) overlapping or duplicate participants.

### 2.4 Data extraction

All retrieved records were imported into EndNote. Two reviewers (Yang and Zhao) independently screened titles and abstracts against the predefined eligibility criteria and then reviewed the full texts of potentially eligible reports. Any discrepancies were resolved through discussion or consultation with a third reviewer (Zhang).

Data were independently extracted by two reviewers (Zhang and Zhao) using a standardized electronic extraction form. Extracted variables included author name, year of publication, study location, intervention and control details, treatment duration, baseline characteristics of participants, and outcome measures.

### 2.5 Quality assessment

The methodological quality of included studies was independently assessed by two reviewers (Yang and Tang) using the Cochrane Risk of Bias 2.0 (ROB 2.0) tool (17). This tool assesses five domains: random sequence generation, allocation concealment, blinding, completeness of outcome data, and selective reporting. Each domain was rated as low risk of bias (RoB), some concerns, or high RoB. The overall risk of bias for a study was considered low if all domains were rated low risk or if only one domain raised some concerns. A study was considered high risk if any domain was rated high or if four or more domains raised some concerns. Studies that did not meet either criterion were classified as having some concerns. Disagreements were resolved through discussion or consultation with a third reviewer (Zhao).

### 2.6 Statistical analysis

All statistical analyses were performed using STATA version 15.1 and R version 4.4.1. Mean differences (MDs) with 95% credible intervals (CrIs) were calculated for continuous outcomes (sex hormone levels), and risk ratios (RRs) with 95% CrIs were calculated for dichotomous outcomes. Statistical heterogeneity was assessed with the Cochrane I^2^ statistic. I^2^ values > 50% indicated substantial heterogeneity. Random-effects models were applied when heterogeneity was present; otherwise, fixed-effects models were used. Publication bias was assessed visually using funnel plots, and *p* < 0.05 denoted the presence of publication bias.

For the network meta-analysis, the assumptions of transitivity, homogeneity, and consistency must be met. Transitivity was assessed qualitatively by comparing baseline characteristics across studies. Homogeneity was tested using the mtc.anohe function in the GeMTC package, with I^2^ < 50% indicating acceptable heterogeneity. Consistency between direct and indirect evidence was examined using node-splitting (mtc.nodesplit function in GeMTC), where *p* > 0.05 indicated no significant inconsistency. Convergence was evaluated using convergence diagnostics, with a Gelman-Rubin statistic of 1 indicating ideal convergence.

Network plots were generated, with nodes representing interventions and edges representing direct comparisons. Node size corresponded to the number of trials per intervention, and edge thickness corresponded to the number of comparisons. Interventions were ranked using the surface under the cumulative ranking curve (SUCRA), representing the probability of each intervention being the most effective. All network graphs were generated using RStudio version 4.1.1.

## 3 Results

### 3.1 Literature search and study selection

**Figure 1:**
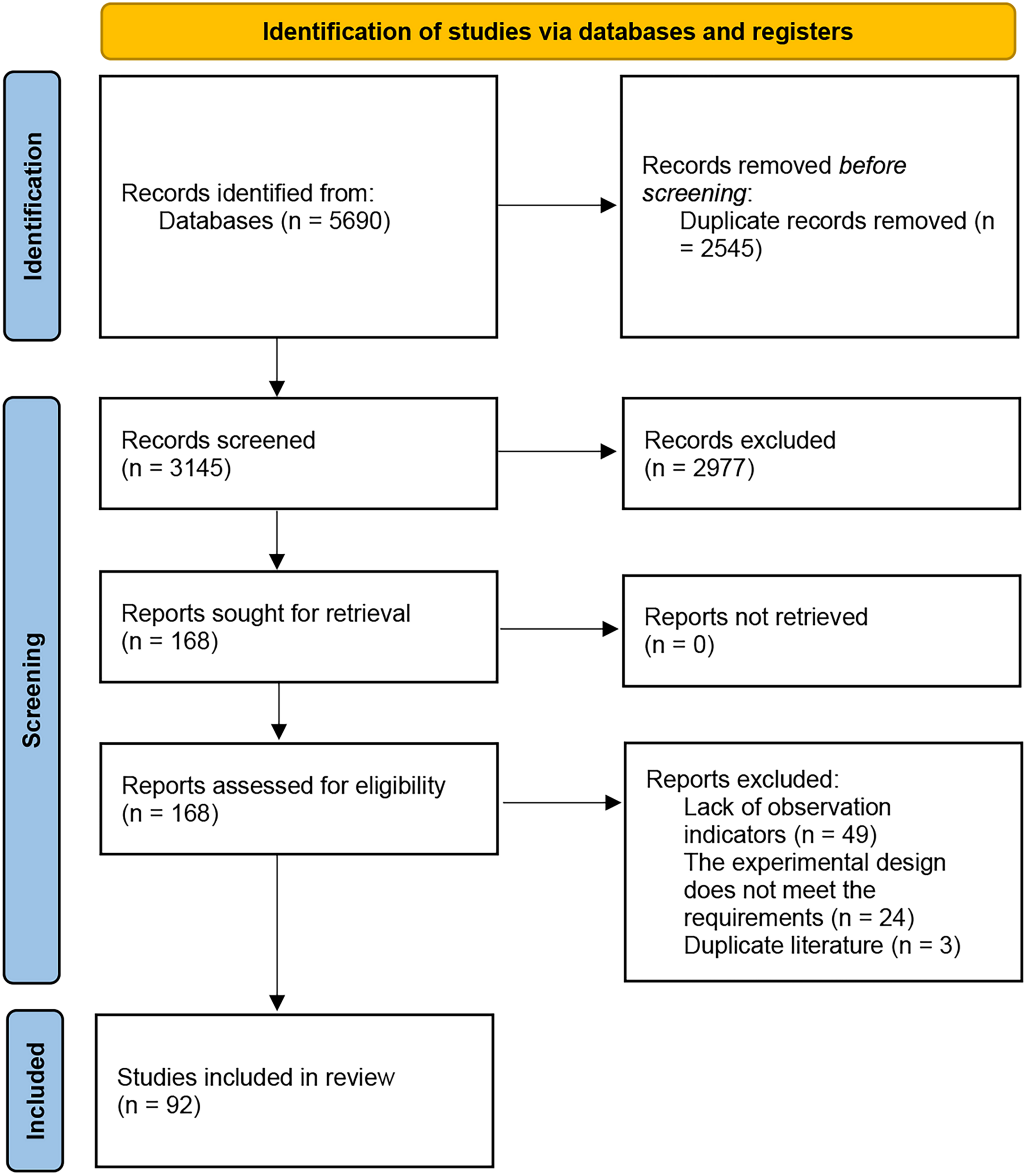
Process of literature identification and selection.

A total of 5,690 articles were initially retrieved from the databases. After removing 2,545 duplicates, 2,977 studies were excluded after checking the titles and abstracts. The full texts of the remaining articles were assessed for eligibility. Ultimately, 92 studies were included in the analysis. The study selection process is illustrated in Figure 1.

### 3.2 Characteristics and quality assessment of included studies

The 92 included studies were RCTs (18–109), comprising 8,165 participants, with 4,108 in the intervention groups and 4,057 in the control groups. Participants were between 17 and 34 years old. Treatment duration ranged from one to nine menstrual cycles, with most studies (72.8%) using three cycles. All trials were conducted in China and published between 2007 and 2024. Baseline characteristics were generally comparable across intervention groups (Table 1 and Supplementary Table 2), suggesting reasonable transitivity within this network. Fourteen acupuncture-related interventions were investigated, including filiform needle (FN) used in 34 studies, CEIA in 19 studies, warming needle moxibustion (WNM) in eight studies, FN + electroacupuncture (FN + E) in eight studies, FN + WNM in five studies, electroacupuncture in three studies, FN + moxibustion (FN + M) in three studies, moxibustion in two studies, FN + WNM + electroacupuncture (FN + WNM + E) in two studies, WNM + electroacupuncture (WNM + E) in two studies, FN + thunder fire moxibustion (FN + TFM) in two studies, acupoint injection in one study, fire needle in one study, FN + ear acupuncture (FN + EA) in one study, and CEIA + moxibustion (CEIA + M) in one study (Supplementary Table 3). Detailed study characteristics are summarized in Table 1 Supplementary Table 2.

**Table 1:** Overview of the studies included in the analysis.

First author	Year	Region	Number of cases		Age		Intervention measures		Course	Number of lost follow-ups		Outcome indicators
			EG	CG	EG	CG	EG	CG		EG	CG	
Chen et al. (21)	2009	China	50	46	31.07 ± 4.46	32.59 ± 3.92	Ovulation-inducing Western medicine + Electroacupuncture	Ovulation-inducing Western medicine	One menstrual cycle	0	4	FSH, LH, E2, pregnancy rate
Li Chen et al. (79)	2019	China	34	34	29.8 ± 3.3	30.5 ± 3.5	Ovulation-inducing Western medicine + Catgut embedment in acupoint	Ovulation-inducing Western medicine	Three menstrual cycles	0	0	FSH, LH, LH/FSH, T, adverse reaction
Wang et al. (107)	2021	China	48	46	28.14 ± 6.45	29.26 ± 6.29	Ovulation-inducing Western medicine + Filiform needle + Warming needle moxibustion	Ovulation-inducing Western medicine	Three menstrual cycles	0	2	LH/FSH, E2, T, overall efficiency
Cui et al. (20)	2012	China	34	32	29.3 ± 3.7	29.3 ± 3.7	Ovulation-inducing Western medicine + Electroacupuncture	Ovulation-inducing Western medicine	N/A	2	0	FSH, LH, E2, P
Dai (82)	2017	China	50	48	29.62 ± 6.49	29.75 ± 6.57	Ovulation-inducing Western medicine + ear acupuncture + Filiform needle	Ovulation-inducing Western medicine	One menstrual cycle	0	0	LH, FSH, pregnancy rate, ovulation rate
Liu et al. (31)	2022	China	52	52	28.12 ± 3.62	28.24 ± 5.41	Ovulation-inducing Western medicine + Filiform needle	Ovulation-inducing Western medicine	Three menstrual cycles	0	0	E2, FSH
Gan and Deng (57)	2022	China	30	30	32.7 ± 4.2	34.5 ± 2.5	Ovulation-inducing Western medicine + Catgut embedment in acupoint + Moxibustion	Ovulation-inducing Western medicine	Three menstrual cycles	0	0	LH/FSH, T, overall efficiency
Zhai (38)	2017	China	40	40	23.7 ± 2.2	23.1 ± 1.9	Ovulation-inducing Western medicine + Catgut embedment in acupoint	Ovulation-inducing Western medicine	Three menstrual cycles	0	0	LH, FSH, T, ovulation rate
Fu et al. (54)	2024	China	45	45	31.41 ± 2.56	27.33 ± 2.41	Ovulation-inducing Western medicine + Catgut embedment in acupoint	Ovulation-inducing Western medicine	One menstrual cycle	0	0	LH, T, FSH, pregnancy rate
Li et al. (68)	2022	China	30	30	33.01 ± 5.92	31.39 ± 6.28	Ovulation-inducing Western medicine + Catgut embedment in acupoint	Ovulation-inducing Western medicine	Three menstrual cycles	0	0	LH, FSH, E2, T
Guo et al. (61)	2021	China	40	40	28.31 ± 2.67	28.21 ± 2.45	Ovulation-inducing Western medicine + Warming needle moxibustion	Ovulation-inducing Western medicine	Three menstrual cycles	0	0	LH, FSH, T, overall efficiency
Guo (49)	2019	China	46	46	27.12 ± 1.25	26.34 ± 2.89	Ovulation-inducing Western medicine + Filiform needle + Electroacupuncture	Ovulation-inducing Western medicine	Three menstrual cycles	0	0	FSH, LH, T, overall efficiency
He et al. (78)	2020	China	62	62	23.21 ± 4.58	23.35 ± 4.26	Ovulation-inducing Western medicine + Catgut embedment in acupoint	Ovulation-inducing Western medicine	Three menstrual cycles	0	0	FSH, LH, T, LH/FSH, overall efficiency, adverse reaction
Jingliu He	2023	China	52	52	29.41 ± 2.31	29.68 ± 2.25	Ovulation-inducing Western medicine + Filiform needle	Ovulation-inducing Western medicine	Three menstrual cycles	0	0	FSH, E2, LH, overall efficiency, adverse reaction
He and Huang (23)	2020	China	35	35	31.97 ± 3.84	32.05 ± 3.63	Ovulation-inducing Western medicine + Electroacupuncture + Warming needle moxibustion	Ovulation-inducing Western medicine	One menstrual cycle	0	0	LH, FSH, LH/FSH, T, overall efficiency
Li et al. (41)	2016	China	14	14	N/A	N/A	Ovulation-inducing Western medicine + Filiform needle	Ovulation-inducing Western medicine	Six menstrual cycles	0	0	LH, FSH, LH/FSH, T
Huang (51)	2021	China	30	30	30.54 ± 6.62	30.89 ± 6.77	Ovulation-inducing Western medicine + Filiform needle	Ovulation-inducing Western medicine	Three menstrual cycles	0	0	LH, FSH, E2, T, overall efficiency, ovulation rate, pregnancy rate
Huang and Wang (36)	2024	China	30	30	27.23 ± 4.96	26.10 ± 5.87	Ovulation-inducing Western medicine + Filiform needle + Warming needle moxibustion	Ovulation-inducing Western medicine	Three menstrual cycles	0	0	T, LH, FSH, LH/FSH, overall efficiency
Huang et al. (77)	2022	China	54	54	28.01 ± 3.14	26.49 ± 2.78	Ovulation-inducing Western medicine + Catgut embedment in acupoint	Ovulation-inducing Western medicine	Three menstrual cycles	0	0	T, LH, FSH, LH/FSH, adverse reaction, pregnancy rate
Shan and Lai (33)	2022	China	30	30	29.40 ± 4.25	29.50 ± 4.11	Ovulation-inducing Western medicine + Warming needle moxibustion	Ovulation-inducing Western medicine	Six menstrual cycles	0	0	LH, FSH, T, E2, overall efficiency, pregnancy rate
Li (67)	2019	China	50	50	27.78 ± 5.96	28.03 ± 5.82	Ovulation-inducing Western medicine + Warming needle moxibustion	Ovulation-inducing Western medicine	Three menstrual cycles	0	0	LH, FSH, E2, overall efficiency
Li et al. (25)	2009	China	23	20	32.63 ± 4.26	32.48 ± 4.45	Ovulation-inducing Western medicine + Electroacupuncture	Ovulation-inducing Western medicine	One menstrual cycle	0	3	FSH, LH, E2
Li et al. (100)	2014	China	53	51	27.1 ± 2.5	25.2 ± 1.8	Ovulation-inducing Western medicine + Filiform needle	Ovulation-inducing Western medicine	Six menstrual cycles	0	2	FSH, LH, LH/FSH, T, adverse reaction, ovulation rate, pregnancy rate
Li (97)	2015	China	75	75	25.1 ± 2.3	24.1 ± 2.2	Ovulation-inducing Western medicine + Filiform needle	Ovulation-inducing Western medicine	Six menstrual cycles	0	0	LH, LH/FSH, T
Li et al. (87)	2021	China	20	20	28.2 ± 3.8	28.1 ± 2.6	Ovulation-inducing Western medicine + Filiform needle	Ovulation-inducing Western medicine	Three menstrual cycles	0	0	FSH, LH, E2, T
Li et al. (29)	2022	China	62	62	28.05 ± 3.15	27.64 ± 3.42	Ovulation-inducing Western medicine + Filiform needle	Ovulation-inducing Western medicine	Three menstrual cycles	0	0	FSH, LH, LH/FSH, T, E2, overall efficiency
Chen et al. (21)	2024	China	58	58	N/A	N/A	Ovulation-inducing Western medicine + Filiform needle + Electroacupuncture	Ovulation-inducing Western medicine	Three menstrual cycles	0	0	LH, FSH, LH/FSH, E2, T, ovulation rate, pregnancy rate
Liu et al. (31)	2018	China	138	140	30 ± 7	31.2 ± 8.1	Ovulation-inducing Western medicine + Filiform needle	Ovulation-inducing Western medicine	Two menstrual cycles	2	0	FSH, LH, E2, T, LH/FSH, ovulation rate, pregnancy rate
Lv et al. (105)	2016	China	100	100	32.00 ± 1.89	33.00 ± 1.76	Ovulation-inducing Western medicine + Filiform needle + Warming needle moxibustion + Electroacupuncture	Ovulation-inducing Western medicine	One menstrual cycle	0	0	FSH, LH, E2, T, LH/FSH, overall efficiency
Ma et al. (37)	2016	China	30	30	23 ± 2	25 ± 4	Ovulation-inducing Western medicine + Filiform needle	Ovulation-inducing Western medicine	Three menstrual cycles	0	0	T, adverse reaction, ovulation rate
Men et al. (84)	2023	China	30	30	29.4 ± 1.8	29.5 ± 1.7	Ovulation-inducing Western medicine + Filiform needle	Ovulation-inducing Western medicine	Three menstrual cycles	0	0	FSH, LH, T, LH/FSH, overall efficiency
Jiang and Meng (73)	2014	China	50	50	25.23 ± 1.45	26.12 ± 1.02	Ovulation-inducing Western medicine + Catgut embedment in acupoint	Ovulation-inducing Western medicine	Three menstrual cycles	0	0	FSH, LH, LH/FSH, E2, T
Peng (88)	2020	China	30	30	28.42 ± 1.32	28.32 ± 1.35	Ovulation-inducing Western medicine + Filiform needle	Ovulation-inducing Western medicine	Three menstrual cycles	0	0	FSH, LH, T, E2
Jiang et al. (66)	2021	China	53	53	29.53 ± 3.48	29.93 ± 3.56	Ovulation-inducing Western medicine + Electroacupuncture + Warming needle moxibustion	Ovulation-inducing Western medicine	Three menstrual cycles	0	0	FSH, LH, T, overall efficiency, adverse reaction
Quan et al. (90)	2021	China	30	30	23.80 ± 6.98	22.97 ± 6.21	Ovulation-inducing Western medicine + Filiform needle	Ovulation-inducing Western medicine	Three menstrual cycles	0	0	LH, FSH, LH/FSH, T, overall efficiency
Ruan et al. (100)	2018	China	29	28	28 ± 4	27 ± 5	Ovulation-inducing Western medicine + Filiform needle + Moxibustion	Ovulation-inducing Western medicine	Three menstrual cycles	0	1	E2, ovulation rate, pregnancy rate
Tang et al. (69)	2015	China	30	30	23.4 ± 12.4	24.2 ± 13.1	Ovulation-inducing Western medicine + Catgut embedment in acupoint	Ovulation-inducing Western medicine	Three menstrual cycles	0	0	LH, FSH, LH/FSH, E2, T
Wang (91)	2015	China	40	40	28.1 ± 2.8	28.9 ± 3.2	Ovulation-inducing Western medicine + Filiform needle	Ovulation-inducing Western medicine	Four menstrual cycles	0	0	FSH, LH, T, E2
Wang (30)	2024	China	41	41	28.45 ± 2.17	28.41 ± 2.13	Ovulation-inducing Western medicine + Filiform needle	Ovulation-inducing Western medicine	Three menstrual cycles	0	0	E2, LH, FSH, T
Dun et al. (40)	2018	China	30	30	16.9 ± 1.58	17 ± 1.82	Ovulation-inducing Western medicine + Filiform needle	Ovulation-inducing Western medicine	Three menstrual cycles	0	0	LH, FSH, T, LH/FSH
Ji et al. (42)	2013	China	40	40	23.3 ± 3.2	29.5 ± 4.9	Ovulation-inducing Western medicine + Filiform needle	Ovulation-inducing Western medicine	Three menstrual cycles	0	0	T, E2, overall efficiency, ovulation rate
Wang et al. (35)	2020	China	30	30	26.43 ± 3.52	26.23 ± 3.48	Ovulation-inducing Western medicine + Filiform needle + Electroacupuncture	Ovulation-inducing Western medicine	Three menstrual cycles	0	0	LH, FSH, E2, T, pregnancy rate
Wang and Yang (80)	2019	China	65	65	28.61 ± 2.75	28.92 ± 2.84	Ovulation-inducing Western medicine + Acupoint injection	Ovulation-inducing Western medicine	Three menstrual cycles	0	0	FSH, LH, T, pregnancy rate
Wang et al. (18)	2022	China	60	60	26.03 ± 6.76	27.24 ± 5.21	Ovulation-inducing Western medicine + Filiform needle	Ovulation-inducing Western medicine	Six menstrual cycles	0	0	FSH, LH, T, overall efficiency
Lv et al. (108)	2007	China	30	30	N/A	N/A	Ovulation-inducing Western medicine + Filiform needle + Electroacupuncture	Ovulation-inducing Western medicine	Three menstrual cycles	0	0	E2, overall efficiency
Wang et al. (32)	2015	China	35	35	N/A	N/A	Ovulation-inducing Western medicine + Moxibustion	Ovulation-inducing Western medicine	Three menstrual cycles	0	0	FSH, LH, LH/FSH, T
Wei et al. (64)	2022	China	60	50	29.32 ± 2.90	30.01 ± 2.45	Ovulation-inducing Western medicine + Warming needle moxibustion	Ovulation-inducing Western medicine	One menstrual cycle	0	10	LH, FSH, T, E2, P, pregnancy rate
Wen et al. (52)	2023	China	42	42	22.18 ± 1.16	21.75 ± 1.09	Ovulation-inducing Western medicine + Filiform needle	Ovulation-inducing Western medicine	Six menstrual cycles	0	0	T, E2, LH, FSH, adverse reaction, ovulation rate, pregnancy rate
Wu et al. (95)	2020	China	56	56	28.64 ± 3.73	29.43 ± 2.97	Ovulation-inducing Western medicine + Filiform needle	Ovulation-inducing Western medicine	Three menstrual cycles	0	0	LH, FSH, T, LH/FSH, adverse reaction
Wu et al. (28)	2021	China	50	50	31 ± 3	31 ± 3	Ovulation-inducing Western medicine + Filiform needle	Ovulation-inducing Western medicine	Three menstrual cycles	0	0	E2, P, LH, FSH, P, T, overall efficiency, pregnancy rate
Wu (50)	2018	China	41	41	29.8 ± 8.1	29.4 ± 8.5	Ovulation-inducing Western medicine + Filiform needle	Ovulation-inducing Western medicine	One menstrual cycle	0	0	FSH, E2, LH, T, overall efficiency
Xiao et al. (71)	2014	China	36	36	22.36 ± 3.22	24.55 ± 3.64	Ovulation-inducing Western medicine + Catgut embedment in acupoint	Ovulation-inducing Western medicine	Three menstrual cycles	0	0	LH, FSH, LH/FSH, T, E2, overall efficiency
Xiao and Wang (44)	2021	China	62	62	31 ± 6	31 ± 6	Ovulation-inducing Western medicine + Fire needle	Ovulation-inducing Western medicine	Three menstrual cycles	0	0	FSH, E2, P, overall efficiency, adverse reaction
Xie and Wang (103)	2019	China	50	50	25.34 ± 6.16	25.32 ± 6.15	Ovulation-inducing Western medicine + Warming needle moxibustion	Ovulation-inducing Western medicine	Three menstrual cycles	0	0	FSH, LH, E2, T, overall efficiency, pregnancy rate, ovulation rate
Zhang et al. (102)	2022	China	60	60	28.52 ± 2.64	29.26 ± 3.27	Ovulation-inducing Western medicine + Warming needle moxibustion	Ovulation-inducing Western medicine	Three menstrual cycles	0	0	LH, T, overall efficiency
Xing et al. (45)	2022	China	36	36	34.85 ± 5.60	34.24 ± 5.67	Ovulation-inducing Western medicine + Filiform needle	Ovulation-inducing Western medicine	Three menstrual cycles	0	0	FSH, LH, E2, T
Xu and Mu (34)	2020	China	41	41	27.73 ± 5.29	28.16 ± 4.39	Ovulation-inducing Western medicine + Filiform needle + Warming needle moxibustion	Ovulation-inducing Western medicine	Nine menstrual cycles	0	0	LH, FSH, T, E2, P, pregnancy rate
Qiu and Xu (106)	2019	China	38	37	29.07 ± 3.27	28.84 ± 3.18	Ovulation-inducing Western medicine + Filiform needle + Warming needle moxibustion	Ovulation-inducing Western medicine	Three menstrual cycles	0	1	P, T, E2, LH, FSH, ovulation rate, pregnancy rate
Xu and Wang (86)	2015	China	38	38	27.51 ± 3.15	27.55 ± 3.16	Ovulation-inducing Western medicine + Filiform needle	Ovulation-inducing Western medicine	Four menstrual cycles	0	0	FSH, LH, E2, T, LH/FSH, overall efficiency, ovulation rate, pregnancy rate
Xu and Zuo (85)	2018	China	30	30	25.7 ± 4.0	25.8 ± 4.2	Ovulation-inducing Western medicine + Filiform needle	Ovulation-inducing Western medicine	Two menstrual cycles	0	0	FSH, LH, E2, T, pregnancy rate, ovulation rate
Xue and Miao (65)	2022	China	40	40	29.45 ± 6.68	27.53 ± 3.81	Ovulation-inducing Western medicine + Filiform needle + Warming needle moxibustion	Ovulation-inducing Western medicine	Three menstrual cycles	0	0	T, LH, FSH, overall efficiency, ovulation rate, pregnancy rate
Li and Xue (26)	2020	China	31	30	N/A	N/A	Ovulation-inducing Western medicine + Filiform needle	Ovulation-inducing Western medicine	Three menstrual cycles	0	1	LH, FSH, LH/FSH, T, overall efficiency
Yan et al. (81)	2018	China	45	45	28.56 ± 7.43	28.67 ± 8.45	Ovulation-inducing Western medicine + Catgut embedment in acupoint	Ovulation-inducing Western medicine	N/A	0	0	LH, FSH, T, E2, P, adverse reaction, pregnancy rate
Yan et al. (75)	2023	China	30	30	27.87 ± 5.23	27.62 ± 5.35	Ovulation-inducing Western medicine + Catgut embedment in acupoint	Ovulation-inducing Western medicine	Three menstrual cycles	0	0	FSH, LH, T, overall efficiency
Yang et al. (19)	2015	China	102	98	31.24 ± 4.72	30.99 ± 4.99	Ovulation-inducing Western medicine + Filiform needle + Electroacupuncture	Ovulation-inducing Western medicine	One menstrual cycle	0	4	LH, E2, P, overall efficiency, pregnancy rate
Yang and Yang (94)	2021	China	39	39	28.61 ± 1.20	28.51 ± 1.18	Ovulation-inducing Western medicine + Filiform needle	Ovulation-inducing Western medicine	Three menstrual cycles	0	0	LH, FSH, T, E2, adverse reaction
Zhang et al. (58)	2016	China	60	60	28.01 ± 2.31	29.45 ± 3.12	Ovulation-inducing Western medicine + Catgut embedment in acupoint	Ovulation-inducing Western medicine	Three menstrual cycles	0	0	FSH, LH, E2, T, P, ovulation rate, pregnancy rate
Yang et al. (63)	2023	China	62	59	27.90 ± 3.15	28.33 ± 4.27	Ovulation-inducing Western medicine + Warming needle moxibustion	Ovulation-inducing Western medicine	Three menstrual cycles	0	3	FSH, T, LH, P
Yang (99)	2018	China	46	45	28.3 ± 4.2	28.3 ± 4.2	Ovulation-inducing Western medicine + Filiform needle	Ovulation-inducing Western medicine	N/A	0	1	T, LH/FSH, LH, FSH
Yang (70)	2016	China	39	39	26.9 ± 3.2	25.8 ± 2.7	Ovulation-inducing Western medicine + Catgut embedment in acupoint	Ovulation-inducing Western medicine	Three menstrual cycles	0	0	FSH, LH, T
Xu et al. (76)	2024	China	74	74	35.62 ± 4.68	34.98 ± 5.01	Ovulation-inducing Western medicine + Catgut embedment in acupoint	Ovulation-inducing Western medicine	Three menstrual cycles	0	0	FSH, LH, T, overall efficiency
Yang (27)	2021	China	58	57	28.13 ± 5.34	28.14 ± 5.29	Ovulation-inducing Western medicine + Filiform needle	Ovulation-inducing Western medicine	Three menstrual cycles	0	1	FSH, LH, E2, T, overall efficiency, pregnancy rate, ovulation rate
Yin et al. (56)	2021	China	53	53	32.75 ± 6.18	33.45 ± 6.29	Ovulation-inducing Western medicine + Filiform needle	Ovulation-inducing Western medicine	Three menstrual cycles	0	0	LH, FSH, LH/FSH, overall efficiency
Yin et al. (43)	2017	China	56	54	28.2 ± 4.1	28.4 ± 3.09	Ovulation-inducing Western medicine + Catgut embedment in acupoint	Ovulation-inducing Western medicine	Three menstrual cycles	0	2	FSH, LH, T, LH/FSH, overall efficiency, ovulation rate, pregnancy rate
Cui et al. (83)	2022	China	28	28	26.53 ± 3.11	26.25 ± 3.01	Ovulation-inducing Western medicine + Filiform needle	Ovulation-inducing Western medicine	Three menstrual cycles	0	0	LH, FSH, E2, P, overall efficiency, ovulation rate
Yu et al. (22)	2018	China	40	40	30 ± 4	29 ± 5	Ovulation-inducing Western medicine + Filiform needle + Electroacupuncture	Ovulation-inducing Western medicine	Three menstrual cycles	0	0	E2, P, overall efficiency, ovulation rate, pregnancy rate
Wang et al. (92)	2021	China	30	29	28.40 ± 5.01	28.52 ± 5.55	Ovulation-inducing Western medicine + Filiform needle + thunder fire moxibustion	Ovulation-inducing Western medicine	Three menstrual cycles	0	1	FSH, LH, T, overall efficiency
Lu et al. (60)	2014	China	20	12	25.64 ± 3.95	24.75 ± 3.87	Ovulation-inducing Western medicine + Catgut embedment in acupoint	Ovulation-inducing Western medicine	Three menstrual cycles	0	8	E2, T, FSH, LH, overall efficiency
Ye and Cheng (48)	2020	China	35	35	26.3 ± 3.9	27.1 ± 3.2	Ovulation-inducing Western medicine + Moxibustion	Ovulation-inducing Western medicine	Six menstrual cycles	0	0	T, LH, E2
Zhang and Li (72)	2020	China	47	44	27 ± 3	26 ± 3	Ovulation-inducing Western medicine + Catgut embedment in acupoint	Ovulation-inducing Western medicine	Three menstrual cycles	0	3	FSH, LH, E2, T, overall efficiency
Zhang (96)	2017	China	64	64	28.3 ± 4.2	27.9 ± 4.3	Ovulation-inducing Western medicine + Filiform needle	Ovulation-inducing Western medicine	Three menstrual cycles	0	0	LH, LH/FSH, T, overall efficiency
Zhang (74)	2022	China	30	30	28.06 ± 5.69	27.80 ± 6.14	Ovulation-inducing Western medicine + Catgut embedment in acupoint	Ovulation-inducing Western medicine	Three menstrual cycles	0	0	T, E2, P, FSH, LH, overall efficiency
Zhang (98)	2016	China	50	50	26.3 ± 4.4	27.2 ± 4.1	Ovulation-inducing Western medicine + Filiform needle	Ovulation-inducing Western medicine	Six menstrual cycles	0	0	FSH, LH, LH/FSH, T
Gu et al. (101)	2018	China	62	62	25.84 ± 4.13	26.11 ± 3.82	Ovulation-inducing Western medicine + Filiform needle	Ovulation-inducing Western medicine	Six menstrual cycles	0	0	E2, FSH, LH, T, P, overall efficiency
Chen et al. (59)	2017	China	30	30	29 ± 6	30 ± 6	Ovulation-inducing Western medicine + Filiform needle + Electroacupuncture	Ovulation-inducing Western medicine	Three menstrual cycles	0	0	E2, FSH, LH, T, ovulation rate, pregnancy rate
Zheng et al. (53)	2024	China	40	40	28.9 ± 2.1	28.8 ± 2.2	Ovulation-inducing Western medicine + Filiform needle	Ovulation-inducing Western medicine	Three menstrual cycles	0	0	FSH, LH, T, overall efficiency, pregnancy rate
Zheng et al. (62)	2022	China	50	50	28.62 ± 4.17	28.49 ± 3.69	Ovulation-inducing Western medicine + Warming needle moxibustion	Ovulation-inducing Western medicine	Three menstrual cycles	0	0	LH, FSH, E2, overall efficiency, pregnancy rate
Zhong et al. (47)	2023	China	31	31	30.31 ± 2.49	30.44 ± 3.19	Ovulation-inducing Western medicine + Filiform needle + thunder fire moxibustion	Ovulation-inducing Western medicine	Three menstrual cycles	0	0	LH, T, overall efficiency
Zhong and Zhang (93)	2019	China	30	30	26.77 ± 4.797	26.57 ± 5.173	Ovulation-inducing Western medicine + Filiform needle + Moxibustion	Ovulation-inducing Western medicine	Three menstrual cycles	0	0	FSH, LH, T, overall efficiency
Zhong et al. (24)	2016	China	30	30	N/A	N/A	Ovulation-inducing Western medicine + Filiform needle + Electroacupuncture	Ovulation-inducing Western medicine	Three menstrual cycles	0	0	LH, LH/FSH, T
Zhu et al. (104)	2015	China	42	40	28.86 ± 4.31	29.02 ± 3.48	Ovulation-inducing Western medicine + Filiform needle + Moxibustion	Ovulation-inducing Western medicine	Three menstrual cycles	0	2	FSH, LH, E2, P, T, ovulation rate, pregnancy rate
Zhuang et al. (39)	2024	China	36	36	29 ± 4	29 ± 3	Ovulation-inducing Western medicine + Filiform needle + Warming needle moxibustion + Electroacupuncture	Ovulation-inducing Western medicine	Three menstrual cycles	0	0	FSH, LH, E2, T, ovulation rate, pregnancy rate

**Figure 2:**
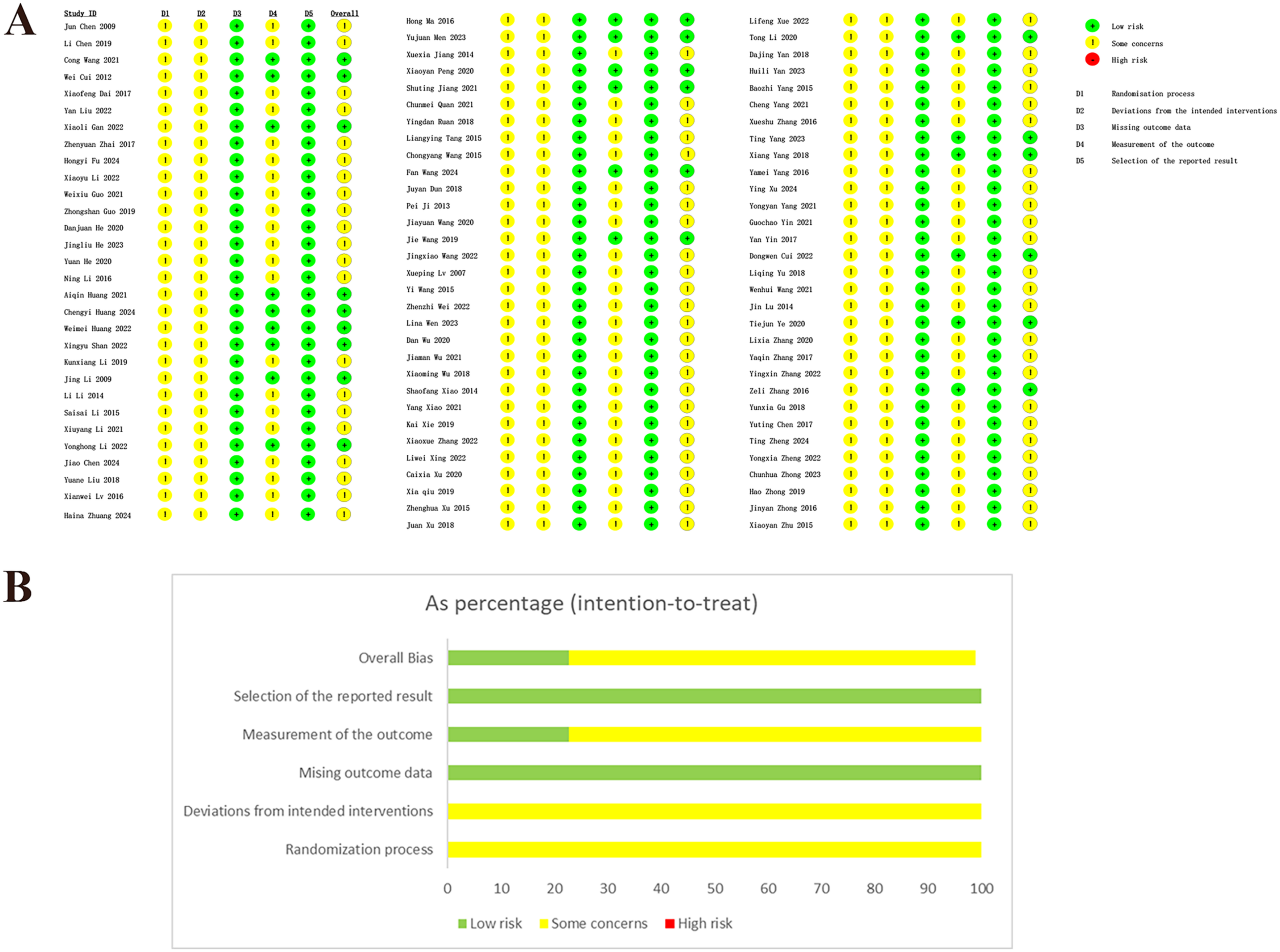
Assessment of the risk of bias in the included studies using the RoB 2.0 tool: **(A)** Distribution across specific domains and the overall risk of bias. **(B)** Bar graph showing the weighted distribution of risk of bias ratings.

The quality of the included studies was appraised using the Cochrane Risk of Bias 2.0 tool. Due to the inherent limitations of acupuncture as a therapeutic method, reliable blinding could not be implemented during treatment. Consequently, none of the RCTs included in this study employed blinding. Moreover, some studies (21, 25, 34, 36, 38, 41, 43, 47, 49, 59, 62, 66, 71–73, 77, 79, 83, 86, 88, 90–92, 97–99, 101, 102, 105, 108, 109) did not describe the methods used to generate randomized controlled cases, which may result in a certain risk of bias. The quality assessment indicated that most studies had a low RoB or some concerns across assessed domains, reflecting generally moderate-to-high methodological quality. Detailed results are presented in Figure 2.

However, certain interventions (e.g., fire needle and auricular acupuncture) were evaluated in only a small number of studies, which may introduce potential publication bias. Sensitivity analyses excluding studies with some concerns or high risk of bias were performed, and the results (presented in Supplementary Table 3) showed that SUCRA rankings remained largely unchanged, supporting the robustness of our findings.

### 3.3 Network diagram

**Figure 3:**
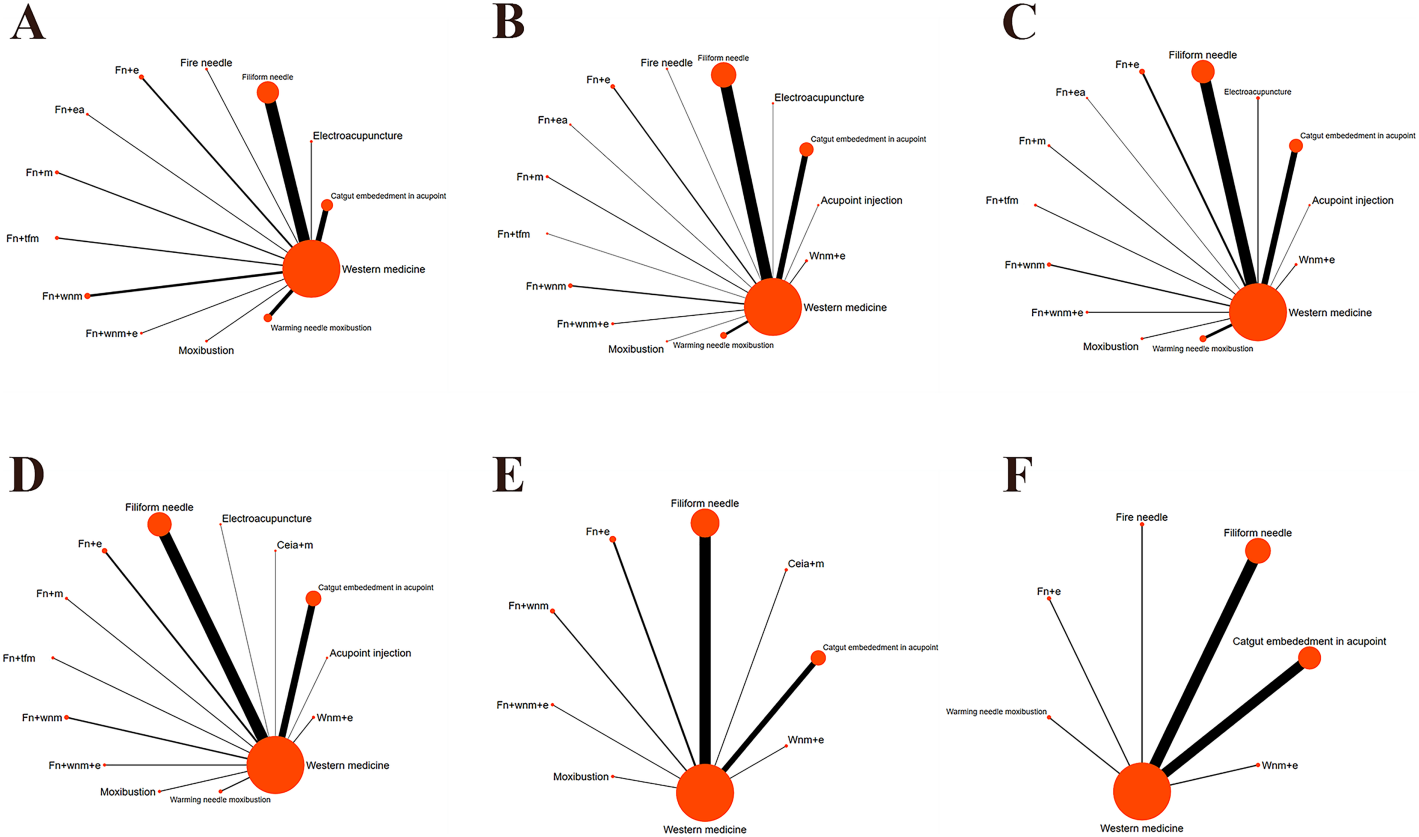
Network diagrams for treatment comparisons: **(A)** TE; **(B)** FSH; **(C)** LH; **(D)** T, **(E)** LH/FSH; **(F)** AE.

In the network plot, nodes represent individual interventions, and node size is proportional to the number of studies on each intervention. Edges indicate direct head-to-head comparisons, and thickness corresponds to the number of studies per comparison (Figure 3). The potential scale reduction factor (PSRF) for all outcomes was 1.00, indicating satisfactory model convergence.

### 3.4 Total efficacy

A total of 49 studies involving 4,455 patients reported TE. With no heterogeneity (*I*^2^ = 0%), a fixed-effects model was applied. Pooled results indicated that electroacupuncture significantly outperformed all other interventions except FN + TFM [Western medicine versus (vs.) electroacupuncture: RR = 0.43; 95% CrI: (0.22, 0.70); FN vs. electroacupuncture: RR = 0.53; 95% CrI: (0.26, 0.85); CEIA vs. electroacupuncture: RR = 0.54; 95% CrI: (0.27, 0.89); WNM vs. electroacupuncture: RR = 0.52; 95% CrI: (0.26, 0.85); electroacupuncture vs. moxibustion: RR = 1.89; 95% CrI: (1.11, 3.90); electroacupuncture vs. fire needle: RR = 2.00; 95% CrI: (1.21, 4.02); electroacupuncture vs. FN + E: RR = 1.79; 95% CrI: (1.08, 3.61); electroacupuncture vs. FN + WNM: RR = 1.82; 95% CrI: (1.11, 3.64); electroacupuncture vs. FN + M: RR = 1.79; 95% CrI: (1.04, 3.68); electroacupuncture vs. FN + WNM + E: RR = 2.00; 95% CrI: (1.22, 4.00); (Table 2)].

**Table 2:** League table for TE.

Intervention											
Western medicine											
**0.82 (0.78, 0.86)**	Filiform needle										
**0.79 (0.74, 0.85)**	0.97 (0.89, 1.05)	Catgut embedment in acupoint									
**0.83 (0.76, 0.89)**	1.01 (0.91, 1.1)	1.04 (0.94, 1.15)	Warming needle moxibustion								
**0.43 (0.22, 0.7)**	**0.53 (0.26, 0.85)**	**0.54 (0.27, 0.89)**	**0.52 (0.26, 0.85)**	Electroacupuncture							
**0.82 (0.64, 0.99)**	1 (0.78, 1.22)	1.03 (0.8, 1.27)	0.99 (0.77, 1.22)	**1.89 (1.11, 3.9)**	Moxibustion						
**0.87 (0.75, 0.97)**	1.06 (0.91, 1.19)	1.09 (0.93, 1.25)	1.05 (0.89, 1.21)	**2 (1.21, 4.02)**	1.06 (0.83, 1.38)	Fire needle					
**0.77 (0.67, 0.88)**	0.94 (0.81, 1.08)	0.98 (0.83, 1.13)	0.94 (0.8, 1.09)	**1.79 (1.08, 3.61)**	0.94 (0.74, 1.24)	0.89 (0.74, 1.08)	FN + E				
**0.78 (0.69, 0.88)**	0.95 (0.84, 1.08)	0.99 (0.86, 1.13)	0.95 (0.82, 1.09)	**1.82 (1.11, 3.64)**	0.96 (0.76, 1.25)	0.91 (0.76, 1.08)	1.01 (0.85, 1.21)	FN + WNM			
**0.73 (0.54, 0.88)**	0.89 (0.65, 1.08)	0.92 (0.67, 1.13)	0.88 (0.65, 1.09)	1.67 (0.95, 3.44)	0.89 (0.62, 1.21)	0.84 (0.61, 1.07)	0.94 (0.68, 1.2)	0.93 (0.67, 1.17)	FN + TFM		
**0.77 (0.6, 0.97)**	0.94 (0.73, 1.19)	0.98 (0.75, 1.24)	0.94 (0.72, 1.2)	**1.79 (1.04, 3.68)**	0.95 (0.69, 1.31)	0.89 (0.68, 1.17)	1 (0.75, 1.31)	0.99 (0.75, 1.28)	1.07 (0.77, 1.55)	FN + M	
**0.86 (0.77, 0.96)**	1.05 (0.93, 1.18)	1.09 (0.95, 1.23)	1.04 (0.91, 1.19)	**2 (1.22, 4)**	1.05 (0.84, 1.37)	1 (0.84, 1.19)	1.11 (0.93, 1.33)	1.1 (0.94, 1.29)	1.18 (0.94, 1.62)	1.11 (0.86, 1.46)	FN + WNM + E

The data in the table are the RR (95%CrI) of the intervention in the column compared to the intervention in the row. A larger RR represents higher efficiency; 1 represents no difference between the two interventions. Bold represents statistically significant.

**Figure 4:**
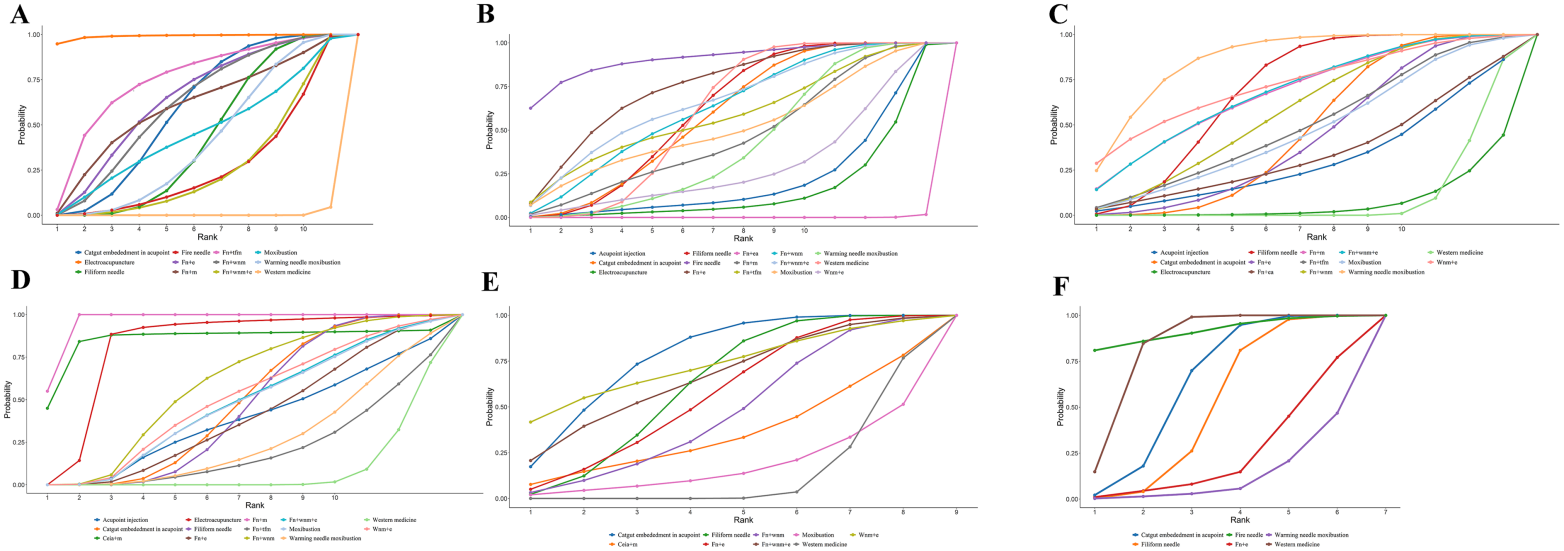
Ranking curves showing cumulative probabilities: **(A)** TE; **(B)** FSH; **(C)** LH; **(D)** T; **(E)** LH/FSH; **(F)** AE.

The SUCRA ranking of TE was as follows: electroacupuncture (99.03%) > FN + TFM (74.77%) > FN + E (64.02%) (Figure 4A and Supplementary Table 4).

Network meta-regression with sample size, mean age, treatment duration, and publication year identified sample size as a significant moderator for filiform needle (*β* = −0.44, 95% CrI: −0.76 to −0.13), with larger studies showing better efficacy, and mean age as a significant moderator for catgut embedment in acupoint (*β* = −1.80, 95% CrI: −3.60 to −0.04). No other significant moderators were found.

### 3.5 Follicle-stimulating hormone (FSH)

Seventy-six studies involving 6,787 participants reported FSH levels. Substantial heterogeneity was observed (*I*^2^ = 85%), and a random-effects model was applied. Pooled results indicated that the FN + EA significantly lowered FSH levels compared to other interventions except electroacupuncture and acupoint injection Western medicine vs. FN + EA: MD = 4.78; 95% CrI: (2.99, 6.57); FN vs. FN + EA: MD = 4.80; 95% CrI: (2.98, 6.61); CEIA vs. FN + EA: MD = 4.76; 95% CrI: (2.91, 6.61); WNM vs. FN + EA: MD = 4.44; 95% CrI: (2.53, 6.35); moxibustion vs. FN + EA: MD = 4.56; 95% CrI: (1.91, 7.20); fire needle vs. FN + EA: MD = 6.06; 95% CrI: (3.50, 8.64); FN + EA vs. FN + E: MD = −5.14; 95% CrI: (−7.18, −3.11); FN + EA vs. FN + WNM: MD = −4.85; 95% CrI: (−6.86, −2.84); FN + EA vs. FN + TFM: MD = −4.78; 95% CrI: (−7.30, −2.26); FN + EA vs. FN + M: MD = −4.47; 95% CrI: (−6.68, −2.24); FN + EA vs. FN + WNM + E: MD = −4.97; 95% CrI: (−7.13, −2.78); FN + EA vs. WNM + E: MD = −3.81; 95% CrI: (−6.41, −1.24). Electroacupuncture and acupoint injection also significantly lowered FSH levels compared to fire needle [electroacupuncture vs. fire needle: MD = −2.96; 95% CrI: (−5.61, −0.31); acupoint injection vs. fire needle: MD = −2.63; 95% CrI: (−5.25, −0.03); (Table 3)].

**Table 3:** League table for FSH.

Intervention														
Western medicine														
−0.01 (−0.34, 0.31)	Filiform needle													
0.02 (−0.44, 0.5)	0.04 (−0.53, 0.62)	Catgut embedment in acupoint												
0.34 (−0.35, 1.03)	0.35 (−0.41, 1.11)	0.32 (−0.52, 1.14)	Warming needle moxibustion											
1.68 (−0.22, 3.58)	1.69 (−0.23, 3.63)	1.66 (−0.3, 3.62)	1.34 (−0.67, 3.37)	Electroacupuncture										
1.35 (−0.5, 3.2)	1.37 (−0.51, 3.25)	1.33 (−0.58, 3.23)	1.01 (−0.96, 2.98)	−0.33 (−2.98, 2.33)	Acupoint injection									
0.22 (−1.72, 2.17)	0.23 (−1.74, 2.22)	0.19 (−1.8, 2.2)	−0.12 (−2.18, 1.95)	−1.46 (−4.19, 1.26)	−1.13 (−3.8, 1.56)	Moxibustion								
−1.28 (−3.13, 0.57)	−1.27 (−3.14, 0.61)	−1.3 (−3.21, 0.6)	−1.62 (−3.59, 0.35)	**−2.96 (−5.61, −0.31)**	**−2.63 (−5.25, −0.03)**	−1.51 (−4.2, 1.19)	Fire needle							
**4.78 (2.99, 6.57)**	**4.8 (2.98, 6.61)**	**4.76 (2.91, 6.61)**	**4.44 (2.53, 6.35)**	**3.1 (0.49, 5.7)**	**3.43 (0.85, 6.01)**	**4.56 (1.91, 7.2)**	**6.06 (3.5, 8.64)**	FN + EA						
−0.36 (−1.33, 0.61)	−0.35 (−1.36, 0.68)	−0.38 (−1.47, 0.7)	−0.7 (−1.88, 0.49)	−2.04 (−4.18, 0.1)	−1.71 (−3.79, 0.38)	−0.58 (−2.76, 1.6)	0.92 (−1.16, 3.01)	**−5.14 (−7.18, −3.11)**	FN + E					
−0.07 (−0.98, 0.85)	−0.06 (−1.02, 0.92)	−0.09 (−1.12, 0.93)	−0.41 (−1.55, 0.74)	−1.75 (−3.86, 0.36)	−1.42 (−3.47, 0.65)	−0.29 (−2.45, 1.86)	1.22 (−0.85, 3.27)	**−4.85 (−6.86, −2.84)**	0.29 (−1.03, 1.62)	FN + WNM				
0 (−1.78, 1.78)	0.01 (−1.8, 1.83)	−0.03 (−1.87, 1.82)	−0.34 (−2.25, 1.57)	−1.68 (−4.29, 0.92)	−1.35 (−3.92, 1.22)	−0.22 (−2.86, 2.43)	1.28 (−1.28, 3.85)	**−4.78 (−7.3, −2.26)**	0.36 (−1.67, 2.39)	0.07 (−1.93, 2.06)	FN + TFM			
0.32 (−1, 1.62)	0.33 (−1.02, 1.67)	0.29 (−1.1, 1.67)	−0.03 (−1.5, 1.45)	−1.36 (−3.69, 0.94)	−1.04 (−3.3, 1.22)	0.09 (−2.26, 2.44)	1.59 (−0.67, 3.86)	**−4.47 (−6.68, −2.24)**	0.67 (−0.95, 2.3)	0.38 (−1.21, 1.98)	0.32 (−1.89, 2.53)	FN + M		
−0.18 (−1.43, 1.07)	−0.17 (−1.45, 1.12)	−0.21 (−1.54, 1.13)	−0.52 (−1.95, 0.91)	−1.87 (−4.14, 0.41)	−1.53 (−3.76, 0.7)	−0.4 (−2.71, 1.91)	1.1 (−1.13, 3.33)	**−4.97 (−7.13, −2.78)**	0.18 (−1.41, 1.75)	−0.11 (−1.67, 1.44)	−0.18 (−2.36, 2)	−0.5 (−2.3, 1.31)	FN + WNM + E	
0.97 (−0.91, 2.82)	0.98 (−0.92, 2.86)	0.95 (−0.99, 2.86)	0.63 (−1.37, 2.61)	−0.71 (−3.37, 1.94)	−0.38 (−3.01, 2.22)	0.75 (−1.96, 3.43)	2.25 (−0.37, 4.87)	**−3.81 (−6.41, −1.24)**	1.33 (−0.78, 3.43)	1.04 (−1.05, 3.11)	0.97 (−1.62, 3.54)	0.66 (−1.63, 2.92)	1.15 (−1.09, 3.38)	WNM + E

The data in the table are the MD (95%CrI) of the intervention in the column compared to the intervention in the row. Bold represents statistically significant.

The SUCRA ranking for FSH levels was as follows: fire needle (90.99%) > FN + E (75.29%) > FN + WNM + E (66.76%) (Figure 4B; Supplementary Table 4).

Network meta-regression identified publication year as a significant moderator for filiform needle (*β* = −1.01, 95% CrI: −1.73 to −0.29), with more recent studies showing greater FSH reduction. No other significant moderators were identified.

### 3.6 Luteinizing hormone

Eighty-three studies involving 7,427 participants reported LH levels. Given minimal heterogeneity (*I*^2^ = 2%), a fixed-effects model was applied. Pooled results indicated that LH concentrations were significantly higher in the Western medicine group compared with multiple acupuncture interventions, including FN, CEIA, WNM, FN + E, FN + WNM, FN + M, FN + WNM + E, and WNM + E [Western medicine vs. FN: MD = 2.33; 95% CrI: (1.82, 2.85); Western medicine vs. CEIA: MD = 1.71; 95% CrI: (1.02, 2.41); Western medicine vs. WNM: MD = 3.02; 95% CrI: (1.98, 4.05); Western medicine vs. FN + E: MD = 1.55; 95% CrI: (0.32, 2.78); Western medicine vs. FN + WNM: MD = 2.03; 95% CrI: (0.60, 3.45); Western medicine vs. FN + M: MD = 2.43; 95% CrI: (0.41, 4.44); Western medicine vs. FN + WNM + E: MD = 2.44; 95% CrI: (0.48, 4.39); Western medicine vs. WNM + E: MD = 2.72; 95% CrI: (0.06, 5.43); (Table 4)].

**Table 4:** League table for LH.

Intervention													
Western medicine													
**2.33 (1.82, 2.85)**	Filiform needle												
**1.71 (1.02, 2.41)**	−0.62 (−1.49, 0.25)	Catgut embedment in acupoint											
**3.02 (1.98, 4.05)**	0.69 (−0.47, 1.84)	**1.31 (0.06, 2.55)**	Warming needle moxibustion										
−0.55 (−2.57, 1.45)	**−2.88 (−4.97, −0.81)**	**−2.27 (−4.4, −0.14)**	**−3.58 (−5.83, −1.32)**	Electroacupuncture									
0.81 (−1.88, 3.48)	−1.52 (−4.26, 1.21)	−0.9 (−3.68, 1.87)	−2.21 (−5.08, 0.65)	1.37 (−1.99, 4.71)	Acupoint injection								
1.61 (−0.45, 3.67)	−0.72 (−2.85, 1.41)	−0.1 (−2.28, 2.07)	−1.41 (−3.73, 0.9)	2.16 (−0.73, 5.04)	0.8 (−2.59, 4.18)	Moxibustion							
0.98 (−1.8, 3.78)	−1.35 (−4.19, 1.49)	−0.73 (−3.6, 2.15)	−2.04 (−5.02, 0.95)	1.54 (−1.91, 4.98)	0.17 (−3.7, 4.06)	−0.63 (−4.09, 2.86)	FN + EA						
**1.55 (0.32, 2.78)**	−0.78 (−2.12, 0.56)	−0.16 (−1.58, 1.26)	−1.47 (−3.08, 0.14)	2.1 (−0.26, 4.46)	0.74 (−2.22, 3.71)	−0.06 (−2.46, 2.34)	0.57 (−2.48, 3.62)	FN + E					
**2.03 (0.6, 3.45)**	−0.3 (−1.82, 1.22)	0.32 (−1.27, 1.91)	−0.99 (−2.75, 0.76)	**2.58 (0.11, 5.05)**	1.21 (−1.81, 4.25)	0.42 (−2.09, 2.93)	1.05 (−2.09, 4.2)	0.48 (−1.41, 2.37)	FN + WNM				
1.71 (−0.31, 3.72)	−0.62 (−2.71, 1.46)	0 (−2.14, 2.13)	−1.31 (−3.59, 0.96)	2.27 (−0.58, 5.12)	0.9 (−2.45, 4.27)	0.11 (−2.78, 2.99)	0.73 (−2.73, 4.17)	0.16 (−2.2, 2.53)	−0.32 (−2.79, 2.16)	FN + TFM			
**2.43 (0.41, 4.44)**	0.1 (−1.99, 2.18)	0.72 (−1.41, 2.85)	−0.59 (−2.86, 1.68)	**2.98 (0.13, 5.82)**	1.62 (−1.74, 4.97)	0.82 (−2.04, 3.7)	1.45 (−2, 4.87)	0.89 (−1.49, 3.24)	0.4 (−2.07, 2.87)	0.72 (−2.14, 3.57)	FN + M		
**2.44 (0.48, 4.39)**	0.11 (−1.92, 2.12)	0.73 (−1.35, 2.8)	−0.58 (−2.79, 1.63)	**2.99 (0.2, 5.8)**	1.63 (−1.69, 4.96)	0.83 (−2, 3.68)	1.46 (−1.97, 4.87)	0.9 (−1.42, 3.19)	0.41 (−2.01, 2.84)	0.73 (−2.08, 3.54)	0.01 (−2.8, 2.81)	FN + WNM + E	
**2.72 (0.06, 5.43)**	0.39 (−2.32, 3.15)	1.01 (−1.74, 3.81)	−0.31 (−3.16, 2.6)	3.27 (−0.05, 6.65)	1.91 (−1.86, 5.72)	1.11 (−2.23, 4.53)	1.74 (−2.1, 5.62)	1.17 (−1.78, 4.14)	0.69 (−2.32, 3.77)	1.01 (−2.32, 4.41)	0.29 (−3.04, 3.66)	0.28 (−3.02, 3.62)	WNM + E

The data in the table are the MD (95%CrI) of the intervention in the column compared to the intervention in the row. Bold represents statistically significant.

The SUCRA-based ranking for LH reduction was as follows: WNM (86.75%) > WNM + E (72.76%) > FN (69.55%) (Figure 4C; Supplementary Table 4).

Network meta-regression with sample size, mean age, treatment duration, and publication year found no significant moderators for LH.

### 3.7 Testosterone

Seventy-two studies involving 6,304 patients reported testosterone levels. Due to moderate heterogeneity (*I*^2^ = 46%), a fixed-effects model was applied. FN + M significantly reduced testosterone levels compared to all other interventions except CEIA + M [Western medicine vs. FN + M: MD = 10.09; 95% CrI: (6.23, 13.81); FN vs. FN + M: MD = 9.59; 95% CrI: (5.73, 13.32); CEIA vs. FN + M: MD = 9.57; 95% CrI: (5.71, 13.30); WNM vs. FN + M: MD = 9.85; 95% CrI: (5.96, 13.62); electroacupuncture vs. FN + M: MD = 8.01; 95% CrI: (3.80, 12.12); acupoint injection vs. FN + M: MD = 9.71; 95% CrI: (5.73, 13.56); moxibustion vs. FN + M: MD = 9.58; 95% CrI: (5.66, 13.37); FN + E vs. FN + M: MD = 9.65; 95% CrI: (5.73, 13.41); FN + WNM vs. FN + M: MD = 9.39; 95% CrI: (5.49, 13.15); FN + TFM vs. FN + M: MD = 9.96; 95% CrI: (6.05, 13.75); FN + M vs. FN + WNM + E: MD = −9.57; 95% CrI: (−13.37, −5.66); FN + M vs. WNM + E: MD = −9.53; 95% CrI: (−13.32, −5.61); (Table 5)].

**Table 5:** League table for T.

Intervention													
Western medicine													
**0.51 (0.27, 0.78)**	0.02 (−0.32, 0.33)	Catgut embedment in acupoint											
0.23 (−0.36, 0.82)	−0.25 (−0.91, 0.34)	−0.28 (−0.94, 0.35)	Warming needle moxibustion										
**2.06 (0.35, 3.79)**	1.57 (−0.17, 3.3)	1.55 (−0.19, 3.29)	**1.83 (0.01, 3.65)**	Electroacupuncture									
0.38 (−0.63, 1.39)	−0.11 (−1.17, 0.89)	−0.13 (−1.19, 0.89)	0.15 (−1.02, 1.31)	−1.68 (−3.68, 0.31)	Acupoint injection								
0.51 (−0.23, 1.23)	0.02 (−0.77, 0.75)	0 (−0.8, 0.76)	0.28 (−0.66, 1.21)	−1.55 (−3.43, 0.32)	0.13 (−1.12, 1.37)	Moxibustion							
0.41 (−0.11, 1.19)	−0.08 (−0.63, 0.66)	−0.1 (−0.67, 0.69)	0.18 (−0.57, 1.18)	−1.64 (−3.43, 0.27)	0.03 (−1.04, 1.36)	0.1 (−0.95, 1.01)	FN + E						
**0.67 (0.13, 1.36)**	0.19 (−0.4, 0.85)	0.17 (−0.44, 0.87)	0.44 (−0.34, 1.36)	−1.38 (−3.18, 0.48)	0.29 (−0.81, 1.55)	0.16 (−0.72, 1.2)	0.26 (−0.61, 1.04)	FN + WNM					
0.12 (−0.57, 0.83)	−0.36 (−1.12, 0.34)	−0.38 (−1.15, 0.35)	−0.11 (−1.02, 0.81)	−1.94 (−3.81, −0.08)	−0.25 (−1.48, 0.97)	0.39 (−1.39, 0.64)	−0.28 (−1.36, 0.55)	−0.55 (−1.55, 0.31)	FN + TFM				
**10.09 (6.23, 13.81)**	**9.59 (5.73, 13.32)**	**9.57 (5.71, 13.3)**	**9.85 (5.96, 13.62)**	**8.01 (3.8, 12.12)**	**9.71 (5.73, 13.56)**	**9.58 (5.66, 13.37)**	**9.65 (5.73, 13.41)**	**9.39 (5.49, 13.15)**	**9.96 (6.05, 13.75)**	FN + M			
0.51 (−0.2, 1.24)	0.03 (−0.75, 0.75)	0.01 (−0.77, 0.76)	0.28 (−0.64, 1.22)	−1.55 (−3.42, 0.32)	0.13 (−1.1, 1.38)	0 (−1.01, 1.04)	0.11 (−0.98, 0.95)	−0.16 (−1.17, 0.72)	0.39 (−0.61, 1.4)	**−9.57 (−13.37, −5.66)**	FN + WNM + E		
0.56 (−0.16, 1.29)	0.07 (−0.71, 0.8)	0.05 (−0.73, 0.81)	0.32 (−0.6, 1.27)	−1.51 (−3.37, 0.37)	0.18 (−1.06, 1.42)	0.05 (−0.97, 1.09)	0.15 (−0.94, 1)	−0.12 (−1.13, 0.77)	0.43 (−0.58, 1.45)	**−9.53 (−13.32, −5.61)**	0.04 (−0.98, 1.06)	WNM + E	
9.17 (−4.45, 23.06)	8.67 (−4.95, 22.57)	8.66 (−4.97, 22.55)	8.93 (−4.7, 22.85)	7.11 (−6.6, 21.12)	8.79 (−4.86, 22.73)	8.66 (−4.98, 22.56)	8.72 (−4.91, 22.63)	8.47 (−5.15, 22.38)	9.05 (−4.6, 22.94)	−0.93 (−14.97, 13.64)	8.66 (−4.98, 22.57)	8.61 (−5.02, 22.51)	CEIA + M

The data in the table are the MD (95%CrI) of the intervention in the column compared to the intervention in the row. Bold represents statistically significant.

The SUCRA ranking for reducing testosterone levels was as follows: FN + M (96.53%) > CEIA + M (85.64%) > electroacupuncture (82.33%) (Figure 4D; Supplementary Table 4).

Network meta-regression with sample size, mean age, treatment duration, and publication year found no significant moderators for testosterone.

### 3.8 LH/FSH ratio

Thirty-three studies involving 3,115 participants reported the LH/FSH ratio. Due to no heterogeneity (*I*^2^ = 0%), a fixed-effects model was applied. The pooled results were as follows: Western medicine vs. FN (MD = 0.55; 95% CrI: 0.36 to 0.74), Western medicine vs. CEIA (MD = 0.68; 95% CrI: 0.40 to 0.97), and Western medicine vs. FN + E (MD = 0.50; 95% CrI: 0.09 to 0.91) (Table 6).

**Table 6:** League table for LH/FSH.

Intervention								
Western medicine								
**0.55 (0.36, 0.74)**	**Filiform needle**							
**0.68 (0.4, 0.97)**	0.14 (−0.21, 0.48)	**Catgut embedment in acupoint**						
−0.1 (−1.05, 0.85)	−0.65 (−1.62, 0.32)	−0.78 (−1.78, 0.21)	**Moxibustion**					
**0.5 (0.09, 0.91)**	−0.05 (−0.5, 0.4)	−0.19 (−0.68, 0.31)	0.6 (−0.44, 1.63)	**FN + E**				
0.4 (−0.08, 0.88)	−0.15 (−0.67, 0.37)	−0.29 (−0.85, 0.27)	0.5 (−0.57, 1.56)	−0.1 (−0.73, 0.53)	**FN + WNM**			
0.61 (−0.08, 1.3)	0.06 (−0.66, 0.77)	−0.07 (−0.82, 0.67)	0.71 (−0.46, 1.88)	0.11 (−0.69, 0.91)	0.21 (−0.63, 1.05)	**FN + WNM + E**		
0.75 (−0.23, 1.72)	0.2 (−0.79, 1.19)	0.06 (−0.95, 1.07)	0.85 (−0.52, 2.2)	0.25 (−0.8, 1.31)	0.35 (−0.73, 1.44)	0.14 (−1.05, 1.33)	**WNM + E**	
0.2 (−0.78, 1.18)	−0.35 (−1.35, 0.65)	−0.48 (−1.5, 0.54)	0.3 (−1.06, 1.67)	−0.3 (−1.36, 0.77)	−0.19 (−1.29, 0.9)	−0.41 (−1.6, 0.79)	−0.55 (−1.93, 0.83)	**CEIA + M**

The data in the table are the MD (95%CrI) of the intervention in the column compared to the intervention in the row. Bold represents statistically significant.

The SUCRA-based ranking for reducing LH/FSH ratio levels was as follows: CEIA (77.73%) > WNM + E (72.87%) > FN + WNM + E (66.39%) (Figure 4E; Supplementary Table 4).

Network meta-regression with sample size, mean age, treatment duration, and publication year found no significant moderators for LH/FSH ratio.

### 3.9 Adverse events

Twenty-one studies involving 1,953 participants reported adverse events. With moderate heterogeneity (*I*^2^ = 29%), a fixed-effects model was applied. The pooled analysis indicated a higher incidence of AEs in the Western medicine group compared with the FN, WNM, and WNM + E groups [Western medicine vs. FN: RR = 1.43; 95% CrI = (1.02, 2.02); Western medicine vs. WNM: RR = 4.77; 95% CrI = (1.18, 35.38); Western medicine vs. WNM + E: RR = 3.17; 95% CrI = (1.16, 11.32); (Table 7)].

**Table 7:** League table for AE.

Intervention						
Western medicine						
**1.43 (1.02, 2.02)**	Filiform needle					
1.21 (0.89, 1.68)	0.85 (0.53, 1.35)	Catgut embedment in acupoint				
**4.77 (1.18, 35.38)**	3.33 (0.79, 25.4)	3.93 (0.94, 29.82)	Warming needle moxibustion			
0.46 (0.06, 2.43)	0.32 (0.04, 1.75)	0.38 (0.05, 2.06)	**0.09 (0.01, 0.86)**	Fire needle		
2.97 (0.88, 13.93)	2.08 (0.58, 10.06)	2.45 (0.69, 11.81)	0.62 (0.06, 4.99)	6.74 (0.81, 82.99)	FN + E	
**3.17 (1.16, 11.32)**	2.22 (0.76, 8.22)	2.61 (0.91, 9.65)	0.66 (0.07, 4.36)	7.13 (0.97, 77.3)	1.07 (0.17, 6.14)	WNM + E

The data in the table are the RR (95%CrI) of the intervention in the column compared to the intervention in the row. A larger RR represents more adverse events, and 1 represents no difference between the two interventions. Bold represents statistically significant.

The SUCRA-based ranking for safety (from least safe to safest) was as follows: fire needle (91.69%) > Western medicine (83.17%) > CEIA (64.08%) (Figure 4F; Supplementary Table 4).

Network meta-regression with sample size, mean age, treatment duration, and publication year found no significant moderators for adverse events.

### 3.10 Publication bias

**Figure 5:**
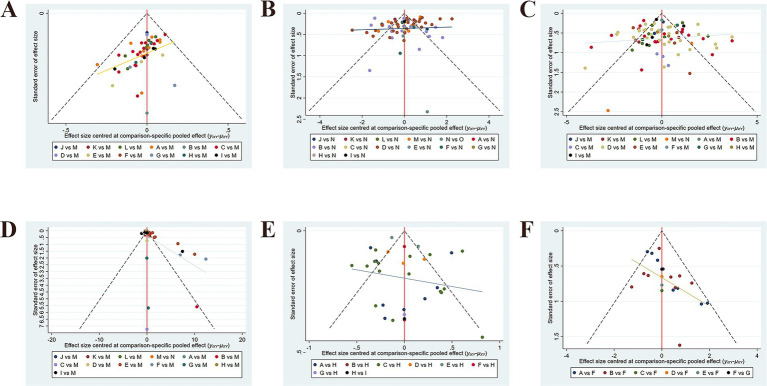
Funnel plots. **(A)** TE; **(B)** FSH; **(C)** LH; **(D)** T; **(E)** LH/FSH; **(F)** AE.

Funnel plots were constructed for outcomes reported in more than 10 studies to assess potential publication bias. As shown in Figure 5, the funnel plot for testosterone levels exhibited asymmetry, suggesting possible publication bias. In contrast, the funnel plots for other outcomes were largely symmetrical, indicating no significant publication bias.

## 4 Discussion

This systematic review and network meta-analysis evaluated the effects of acupuncture and its combined modalities on regulating sex hormone levels in patients with PCOS. A total of 92 RCTs encompassing 8,165 participants and 15 acupuncture-related interventions were included: FN, CEIA, WNM, electroacupuncture, acupoint injection, moxibustion, fire needle, FN + EA, FN + E, FN + WNM, FN + TFM, FN + M, FN + WNM + E, WNM + E, and CEIA + M.

Due to the variety of acupuncture techniques and a lack of direct head-to-head comparisons, it is challenging for clinicians to identify the most effective modalities. Hence, promoting acupuncture in clinical practice and selecting optimal treatment strategies are difficult. To address this limitation, the present study employed a network meta-analysis integrating direct and indirect evidence to compare the efficacy of multiple interventions, providing a robust, evidence-based framework to enhance the generalizability of acupuncture in clinical practice.

The analysis focused on key reproductive hormones (FSH, LH, testosterone levels, and LH/FSH ratio), total efficacy, and the incidence of AEs to explore the clinical effectiveness of acupuncture in PCOS patients.

### 4.1 Sex hormones

Sex hormones serve as critical biomarkers for the diagnosis and treatment of PCOS (110). The primary hormones implicated in PCOS include FSH, LH, testosterone, and the LH/FSH ratio (111). FSH and LH, key pituitary gonadotropins, not only facilitate follicular growth and ovulation but also regulate cyclic endometrial changes.

PCOS is often characterized by elevated LH levels and relatively reduced FSH levels, which may result from increased gonadotropin-releasing hormone (GnRH) pulse frequency. Clinically, lower LH levels and higher FSH levels often suggest more favorable treatment responses in patients with PCOS (112). The LH/FSH ratio is particularly informative for the treatment of PCOS, and a LH/FSH ratio ≥2 is frequently observed in non-obese PCOS patients (113–115). Testosterone, an endogenous androgen primarily produced by the ovaries and adrenal glands (116), plays a central role in the pathophysiology of PCOS and serves as a pivotal indicator of hyperandrogenemia. Greater reductions in serum testosterone levels are closely associated with higher therapeutic efficacy (117).

### 4.2 Mechanisms of acupuncture

Hyperandrogenemia, a typical endocrine disturbance in PCOS, is primarily manifested as abnormal increases in circulating free testosterone (118). Excess testosterone can be converted into estrogen in peripheral tissues, including adipose tissue (119). This aberrant hormonal conversion, coupled with impaired progesterone feedback, enhances the activity of gonadotropin-releasing hormone (GnRH) neurons in the hypothalamus (120). GnRH regulates the secretion of LH and FSH by the anterior pituitary gland (121). Abnormally increased pulsatile GnRH secretion can elevate LH levels while relatively suppressing FSH secretion, disrupting local ovarian hormone balance, and ultimately leading to pathological manifestations such as ovulatory dysfunction (122).

Although ovulation-inducing agents are commonly employed in the management of PCOS (123), Western medicine treatment alone is often related to high recurrence rates and suboptimal ovulation outcomes and is ineffective in mitigating long-term complications such as weight gain and hirsutism (124). Growing evidence (125–128) indicates that acupuncture ameliorates endocrine dysfunction in PCOS via multiple mechanisms. On the one hand, acupuncture regulates the hypothalamic–pituitary–ovarian axis, suppresses abnormal secretion of GnRH, and modulates the opioid peptide system to influence the release of gonadotropin (129). On the other hand, it improves ovarian blood flow and neural regulation, thereby promoting restoration of morphology and function of the ovary (130). The present network meta-analysis demonstrated that acupuncture and its combination therapies significantly reduced LH, the LH/FSH ratio, and testosterone levels, supporting their efficacy in restoring hormonal balance in PCOS patients.

Nonetheless, acupuncture may induce adverse effects. Reported complications include organ or tissue injury, local irritation, systemic reactions, infections, burns, and needle breakage (131). Variations in needling technique, electrical stimulation parameters, and moxibustion heat application may contribute to these adverse events, potentially influencing therapeutic outcomes.

### 4.3 Result analysis

Regarding TE, the top five acupuncture interventions were electroacupuncture, FN + TFM, FN + E, FN + WNM, and FN + M, suggesting that electrical and thermal stimulation confer notable therapeutic benefits for PCOS. This may be attributed to the high electrical and thermal sensitivity of acupoints in PCOS, enhancing treatment efficacy (132). Mechanistically, electroacupuncture has been shown to improve ovarian function by modulating immune-inflammatory responses and macrophage polarization (133). Furthermore, electroacupuncture can restore the density and coverage of GnRH axon projections, thereby ameliorating neuroendocrine dysfunction, which is not observed with traditional FN therapy (125). Moxibustion similarly improves androgen levels and ovarian morphology (134) and regulates insulin resistance and gut microbiota, an effect not observed with traditional FN alone (135).

PCOS patients typically present with elevated LH and testosterone levels, an increased LH/FSH ratio, and normal or slightly reduced FSH. Auricular acupuncture, regardless of needling or seed embedding, had a small impact on FSH and LH, likely due to the anatomical distance of auricular acupoints from the hypothalamic–pituitary–gonadal axis. SUCRA rankings indicated that fire needle (90.99%) and FN + E (75.29%) were most effective in modulating FSH. Both strategies involve acupuncture combined with electrical or thermal stimulation, consistent with the aforementioned mechanisms. For LH, WNM (86.75%) and WNM + E (72.76%) ranked highest. For reducing testosterone levels, FN + M (96.53%) ranked first, followed by CEIA + M (85.64%) and electroacupuncture (82.33%). CEIA, as an acupuncture technique, provided sustained stimulation benefits relative to FN. For the LH/FSH ratio, CEIA (77.73%), WNM + E (72.87%), and FN + WNM + E (66.39%) were most effective. Although reported in only 33 studies, acupuncture combined with electrical stimulation or thermal stimulation appeared to be particularly advantageous. Notably, CEIA was the most efficacious, possibly since the LH/FSH level is more sensitive to continuous stimulation.

AEs were analyzed in 21 studies. SUCRA-based rankings suggested a higher risk of AEs with fire needle (91.69%), Western medicine (83.17%), and CEIA (64.08%), which may be related to the relatively strong stimulatory effects of fire needle and CEIA.

Meta-regression further revealed that the efficacy of filiform needle improved with larger sample sizes and more recent publication years for FSH, suggesting that earlier studies may have underestimated its hormonal regulatory effect due to insufficient statistical power. The negative association between age and CEIA efficacy in total effectiveness may reflect diminished treatment response in older PCOS patients. However, the absence of significant moderators for LH, testosterone, LH/FSH ratio, and adverse events indicates that the observed heterogeneity in these outcomes cannot be readily explained by the examined covariates, underscoring the need for standardized protocols in future trials.

### 4.4 Conclusion and future directions

In summary, this network meta-analysis does not support a clear universal superiority of either single or combined acupuncture therapies for PCOS across all outcomes. However, interventions involving electrical stimulation, thermal stimulation, or sustained stimulation tended to show more favorable rankings for total efficacy and sex hormone regulation. This study provides a comparative ranking across 15 acupuncture-related interventions, which may help clinicians select treatment strategies according to specific therapeutic goals and safety considerations. Given the methodological limitations and the exclusively Chinese study populations, these findings should be interpreted cautiously and confirmed in high-quality, multicenter, ethnically diverse RCTs.

Several limitations should be acknowledged. First, all included studies were conducted in Chinese populations. This may limit the generalizability of our findings not only because of differences in cultural context and medical practice but also because PCOS is a heterogeneous disorder with potential ethnic, genetic, and familial influences on clinical phenotype and treatment response. Therefore, caution is warranted when extrapolating these findings to other populations. Second, the number of studies on certain interventions (e.g., fire needle and auricular acupuncture) was small, which may influence the stability of ranking results. Third, many trials did not report randomization and blinding procedures, which may introduce potential bias. Fourth, due to the lack of long-term follow-up data, it is not possible to evaluate the long-term efficacy and safety of the treatment, such as the maintenance of hormone levels and the prevention of long-term complications. Finally, this study did not differentiate the acupuncture point selection schemes of each included trial. The selection of acupuncture points is considered to be an important factor influencing the efficacy of acupuncture and the research results. Therefore, it is very likely to be one of the sources of heterogeneity in this network meta-analysis. Additionally, since all included interventions were acupuncture-related therapies, variations in acupoint selection and needling techniques across studies introduce uncertainty in the interpretation of SUCRA rankings, which should be treated with caution.

Future research should address these gaps. First, conducting rigorously designed, large-scale, multicenter randomized controlled trials is necessary to directly compare key interventions such as electroacupuncture, moxibustion, and acupoint embedment. Stratified analyses by regions, ethnicity, and PCOS phenotype are warranted to identify populations most likely to benefit from acupuncture. Investigating the effect of acupuncture in preventing chronic PCOS-associated complications, including metabolic and cardiovascular disorders, is also recommended. The long-term efficacy of acupuncture for PCOS should also be considered, such as its sustained regulatory effects on sex hormones and its potential role in preventing complications. Finally, integrating multi-omics approaches (such as metabolomics and gut microbiome profiling) may elucidate underlying mechanisms of interventions such as electroacupuncture, moxibustion, and CEIA, providing a theoretical foundation for precision medicine strategies.

## Data Availability

The original contributions presented in the study are included in the article/Supplementary material, further inquiries can be directed to the corresponding author/s.
