# Supplementary material for: Efficacy of acupuncture therapies for polycystic ovary syndrome: a network meta-analysis
Source: Front Med (Lausanne). 2026 Jul 29;13:1895421. doi: 10.3389/fmed.2026.1895421 (PMC13461588; doi:10.3389/fmed.2026.1895421)
Supplement: Supplementary file 1 [file Table_1.DOCX]

**Table S1.** **The search strategy**

**The search strategy (Pubmed)**

| Search number | Query | Results |
| --- | --- | --- |
| 1 | Acupuncture Therapy[MeSH Terms] | 30627 |
| 2 | Electroacupuncture[MeSH Terms] | 5311 |
| 3 | Moxibustion[MeSH Terms] | 2951 |
| 4 | auricular acupuncture[MeSH Terms] | 525 |
| 5 | "abdominal needle"OR"ACET acupoint catgut embedding therapy"OR"acupoint catgut embedding"OR"acupoint catgut embedding therapy"OR"acupoint injection"OR"Acupotom*"OR"acupuncture"OR"Acupuncture Therapy"OR"Acupuncture Treatmen*"OR"auricular acupunctur*"OR"auriculo acupuncture"OR"auriculoacupuncture"OR"auriculotherapy"OR"catgut embedding"OR"catgut implantation"OR"Ear Acupunctur*"OR"earlobe acupuncture"OR"electric acupuncture"OR"electrical acupoint stimulation"OR"electrical acupuncture"OR"electro acupuncture"OR"Electroacupuncture"OR"electrode acupuncture"OR"electronic acupuncture"OR"Moxabustion"OR"Moxibustion"OR"Pharmacoacupuncture Therapy"OR"Pharmacoacupuncture Treatment"OR"shonishin"OR"warming needle moxibustion"[Title/Abstract] | 46059 |
| 6 | Polycystic Ovary Syndrome[MeSH Terms] | 19084 |
| 7 | "cystic ovary"OR"micropolycystic ovary"OR"multiple follicle cyst"OR"ovary polycystic disease"OR"ovary polycystic syndrome"OR"polycystic ovarian disease"OR"Polycystic Ovarian Syndrome"OR"polycystic ovary"OR"polycystic ovary disease"OR"Polycystic Ovary Syndrome"OR"Sclerocystic Ovar*"OR"Sclerocystic Ovarian Degeneration"OR"Sclerocystic Ovary Syndrome"OR"stein cohen leventhal syndrome"OR"stein leventhal disease"OR"Stein Leventhal Syndrome"OR"syndrome stein leventhal"[Title/Abstract] | 26021 |
| 8 | (#1 OR #2 OR #3 OR #4 OR #5) AND (#6 OR #7) | 324 |

**The search strategy (Embase)**

| Search number | Query | Results |
| --- | --- | --- |
| 1 | 'acupuncture'/exp | 60770 |
| 2 | 'electroacupuncture'/exp | 10125 |
| 3 | 'moxibustion'/exp | 5089 |
| 4 | 'auricular acupuncture'/exp | 952 |
| 5 | 'catgut embedding'/exp | 226 |
| 6 | 'abdominal needle':ab,ti,kw OR 'acet acupoint catgut embedding therapy':ab,ti,kw OR 'acupoint catgut embedding':ab,ti,kw OR 'acupoint catgut embedding therapy':ab,ti,kw OR 'acupoint injection':ab,ti,kw OR 'acupotom*':ab,ti,kw OR 'acupuncture':ab,ti,kw OR 'acupuncture therapy':ab,ti,kw OR 'acupuncture treatmen*':ab,ti,kw OR 'auricular acupunctur*':ab,ti,kw OR 'auriculo acupuncture':ab,ti,kw OR 'auriculoacupuncture':ab,ti,kw OR 'auriculotherapy':ab,ti,kw OR 'catgut embedding':ab,ti,kw OR 'catgut implantation':ab,ti,kw OR 'ear acupunctur*':ab,ti,kw OR 'earlobe acupuncture':ab,ti,kw OR 'electric acupuncture':ab,ti,kw OR 'electrical acupoint stimulation':ab,ti,kw OR 'electrical acupuncture':ab,ti,kw OR 'electro acupuncture':ab,ti,kw OR 'electroacupuncture':ab,ti,kw OR 'electrode acupuncture':ab,ti,kw OR 'electronic acupuncture':ab,ti,kw OR 'moxabustion':ab,ti,kw OR 'moxibustion':ab,ti,kw OR 'pharmacoacupuncture therapy':ab,ti,kw OR 'pharmacoacupuncture treatment':ab,ti,kw OR 'shonishin':ab,ti,kw OR 'warming needle moxibustion':ab,ti,kw | 48627 |
| 7 | 'ovary polycystic disease'/exp | 40421 |
| 8 | 'cystic ovary':ab,ti,kw OR 'micropolycystic ovary':ab,ti,kw OR 'multiple follicle cyst':ab,ti,kw OR 'ovary polycystic disease':ab,ti,kw OR 'ovary polycystic syndrome':ab,ti,kw OR 'polycystic ovarian disease':ab,ti,kw OR 'polycystic ovarian syndrome':ab,ti,kw OR 'polycystic ovary':ab,ti,kw OR 'polycystic ovary disease':ab,ti,kw OR 'polycystic ovary syndrome':ab,ti,kw OR 'sclerocystic ovar*':ab,ti,kw OR 'sclerocystic ovarian degeneration':ab,ti,kw OR 'sclerocystic ovary syndrome':ab,ti,kw OR 'stein cohen leventhal syndrome':ab,ti,kw OR 'stein leventhal disease':ab,ti,kw OR 'stein leventhal syndrome':ab,ti,kw OR 'syndrome stein leventhal':ab,ti,kw | 33102 |
| 9 | (#1 OR #2 OR #3 OR #4 OR #5 OR #6) AND (#7 OR #8) | 451 |

**The search strategy (web of science)**

| Search number | Query | Results |
| --- | --- | --- |
| 1 | TS=((abdominal needle) OR (ACET acupoint catgut embedding therapy) OR (acupoint catgut embedding) OR (acupoint catgut embedding therapy) OR (acupoint injection) OR (Acupotom*) OR (acupuncture) OR (Acupuncture Therapy) OR (Acupuncture Treatmen*) OR (auricular acupunctur*) OR (auriculo acupuncture) OR (auriculoacupuncture) OR (auriculotherapy) OR (catgut embedding) OR (catgut implantation) OR (Ear Acupunctur*) OR (earlobe acupuncture) OR (electric acupuncture) OR (electrical acupoint stimulation) OR (electrical acupuncture) OR (electro acupuncture) OR (Electroacupuncture) OR (electrode acupuncture) OR (electronic acupuncture) OR (Moxabustion) OR (Moxibustion) OR (Pharmacoacupuncture Therapy) OR (Pharmacoacupuncture Treatment) OR (shonishin) OR (warming needle moxibustion)) | 37010 |
| 2 | TS=((cystic ovary) OR (micropolycystic ovary) OR (multiple follicle cyst) OR (ovary polycystic disease) OR (ovary polycystic syndrome) OR (polycystic ovarian disease) OR (Polycystic Ovarian Syndrome) OR (polycystic ovary) OR (polycystic ovary disease) OR (Polycystic Ovary Syndrome) OR (Sclerocystic Ovar*) OR (Sclerocystic Ovarian Degeneration) OR (Sclerocystic Ovary Syndrome) OR (stein cohen leventhal syndrome) OR (stein leventhal disease) OR (Stein Leventhal Syndrome) OR (syndrome stein leventhal)) | 33422 |
| 3 | #2 AND #1 | 294 |

**The search strategy (Cochrane Library)**

| Search number | Query | Results |
| --- | --- | --- |
| 1 | MeSH descriptor: [Acupuncture Therapy] explode all trees | 7142 |
| 2 | MeSH descriptor: [Electroacupuncture] explode all trees | 1175 |
| 3 | MeSH descriptor: [Moxibustion] explode all trees | 685 |
| 4 | MeSH descriptor: [Acupuncture, Ear] explode all trees | 266 |
| 5 | ('abdominal needle' OR 'ACET acupoint catgut embedding therapy' OR 'acupoint catgut embedding' OR 'acupoint catgut embedding therapy' OR 'acupoint injection' OR 'Acupotom*' OR 'acupuncture' OR 'Acupuncture Therapy' OR 'Acupuncture Treatmen*' OR 'auricular acupunctur*' OR 'auriculo acupuncture' OR 'auriculoacupuncture' OR 'auriculotherapy' OR 'catgut embedding' OR 'catgut implantation' OR 'Ear Acupunctur*' OR 'earlobe acupuncture' OR 'electric acupuncture' OR 'electrical acupoint stimulation' OR 'electrical acupuncture' OR 'electro acupuncture' OR 'Electroacupuncture' OR 'electrode acupuncture' OR 'electronic acupuncture' OR 'Moxabustion' OR 'Moxibustion' OR 'Pharmacoacupuncture Therapy' OR 'Pharmacoacupuncture Treatment' OR 'shonishin' OR 'warming needle moxibustion'):ab,ti,kw | 24651 |
| 6 | MeSH descriptor: [Polycystic Ovary Syndrome] explode all trees | 2164 |
| 7 | ('cystic ovary' OR 'micropolycystic ovary' OR 'multiple follicle cyst' OR 'ovary polycystic disease' OR 'ovary polycystic syndrome' OR 'polycystic ovarian disease' OR 'Polycystic Ovarian Syndrome' OR 'polycystic ovary' OR 'polycystic ovary disease' OR 'Polycystic Ovary Syndrome' OR 'Sclerocystic Ovar*' OR 'Sclerocystic Ovarian Degeneration' OR 'Sclerocystic Ovary Syndrome' OR 'stein cohen leventhal syndrome' OR 'stein leventhal disease' OR 'Stein Leventhal Syndrome' OR 'syndrome stein leventhal'):ab,ti,kw | 5309 |
| 8 | (#1 OR #2 OR #3 OR #4 OR #5) AND (#6 OR #7) | 222 |

**The search strategy (中国知网)**

| Search number | Query | Results |
| --- | --- | --- |
| 1 | （主题：针灸+温针灸+电针+艾灸+耳穴+穴位埋线+穴位注射+火针+头针+穴位贴敷+皮内针+微针+针刺+脐灸+经皮穴位电刺激+体针+头皮针+腕踝针+激光针灸）AND（主题：多囊卵巢综合征+多囊卵巢+多囊+多囊综合征） | 843 |

**The search strategy (万方)**

| Search number | Query | Results |
| --- | --- | --- |
| 1 | （主题：针灸or温针灸or电针or艾灸or耳穴or穴位埋线or穴位注射or火针or头针or穴位贴敷or皮内针or微针or针刺or脐灸or经皮穴位电刺激or体针or头皮针or腕踝针or激光针灸）and（主题：多囊卵巢综合征or多囊卵巢or多囊or多囊综合征） | 1367 |

**The search strategy (维普)**

| Search number | Query | Results |
| --- | --- | --- |
| 1 | （主题：针灸OR温针灸OR电针OR艾灸OR耳穴OR穴位埋线OR穴位注射OR火针OR头针OR穴位贴敷OR皮内针OR微针OR针刺OR脐灸OR经皮穴位电刺激OR体针OR头皮针OR腕踝针OR激光针灸）AND（主题：多囊卵巢综合征OR多囊卵巢OR多囊OR多囊综合征） | 1127 |

**The search strategy (中国生物医学)**

| Search number | Query | Results |
| --- | --- | --- |
| 1 | （针灸OR温针灸OR电针OR艾灸OR耳穴OR穴位埋线OR穴位注射OR火针OR头针OR穴位贴敷OR皮内针OR微针OR针刺OR脐灸OR经皮穴位电刺激OR体针OR头皮针OR腕踝针OR激光针灸）AND（主题：多囊卵巢综合征OR多囊卵巢OR多囊OR多囊综合征） | 1062 |
