# Supplementary material for: Efficacy of acupuncture therapies for polycystic ovary syndrome: a network meta-analysis
Source: Front Med (Lausanne). 2026 Jul 29;13:1895421. doi: 10.3389/fmed.2026.1895421 (PMC13461588; doi:10.3389/fmed.2026.1895421)
Supplement: Supplementary file 2 [file Table_2.DOCX]

**Table S2** Overview of the studies included in the analysis

| **First author** | **year** | **Region** | **Number of cases** | | **Age** | | **Intervention measures** | | **course** | **Number of lost follow-ups** | | **Outcome indicators** |
| --- | --- | --- | --- | --- | --- | --- | --- | --- | --- | --- | --- | --- |
|  |  |  | **EG** | **CG** | **EG** | **CG** | **EG** | **CG** |  | **EG** | **CG** |  |
| Jun Chen | 2009 | China | 50 | 46 | 31.07±4.46 | 32.59±3.92 | Ovulation-inducing Western medicine+Electroacupuncture | Ovulation-inducing Western medicine | One menstrual cycle | 0 | 4 | FSH, LH, E2, Pregnancy rate |
| Li Chen | 2019 | China | 34 | 34 | 29.8±3.3 | 30.5±3.5 | Ovulation-inducing Western medicine+Catgut embededment in acupoint | Ovulation-inducing Western medicine | Three menstrual cycles | 0 | 0 | FSH, LH, LH/FSH, T, Adverse reaction |
| Cong Wang | 2021 | China | 48 | 46 | 28.14±6.45 | 29.26±6.29 | Ovulation-inducing Western medicine+Filiform needle+Warming needle moxibustion | Ovulation-inducing Western medicine | Three menstrual cycles | 0 | 2 | LH/FSH, E2, T, Overall efficiency |
| Wei Cui | 2012 | China | 34 | 32 | 29.3±3.7 | 29.3±3.7 | Ovulation-inducing Western medicine+Electroacupuncture | Ovulation-inducing Western medicine | N/A | 2 | 0 | FSH, LH, E2, P |
| Xiaofeng Dai | 2017 | China | 50 | 48 | 29.62±6.49 | 29.75±6.57 | Ovulation-inducing Western medicine+ear acupuncture+Filiform needle | Ovulation-inducing Western medicine | One menstrual cycle | 0 | 0 | LH, FSH, Pregnancy rate, Ovulation rate |
| Yan Liu | 2022 | China | 52 | 52 | 28.12±3.62 | 28.24±5.41 | Ovulation-inducing Western medicine+Filiform needle | Ovulation-inducing Western medicine | Three menstrual cycles | 0 | 0 | E2, FSH |
| Xiaoli Gan | 2022 | China | 30 | 30 | 32.7±4.2 | 34.5±2.5 | Ovulation-inducing Western medicine+Catgut embededment in acupoint+Moxibustion | Ovulation-inducing Western medicine | Three menstrual cycles | 0 | 0 | LH/FSH, T, Overall efficiency |
| Zhenyuan Zhai | 2017 | China | 40 | 40 | 23.7±2.2 | 23.1±1.9 | Ovulation-inducing Western medicine+Catgut embededment in acupoint | Ovulation-inducing Western medicine | Three menstrual cycles | 0 | 0 | LH, FSH, T, Ovulation rate |
| Hongyi Fu | 2024 | China | 45 | 45 | 31.41±2.56 | 27.33±2.41 | Ovulation-inducing Western medicine+Catgut embededment in acupoint | Ovulation-inducing Western medicine | One menstrual cycle | 0 | 0 | LH, T, FSH, Pregnancy rate |
| Xiaoyu Li | 2022 | China | 30 | 30 | 33.01±5.92 | 31.39±6.28 | Ovulation-inducing Western medicine+Catgut embededment in acupoint | Ovulation-inducing Western medicine | Three menstrual cycles | 0 | 0 | LH, FSH, E2, T |
| Weixiu Guo | 2021 | China | 40 | 40 | 28.31±2.67 | 28.21±2.45 | Ovulation-inducing Western medicine+Warming needle moxibustion | Ovulation-inducing Western medicine | Three menstrual cycles | 0 | 0 | LH, FSH, T, Overall efficiency |
| Zhongshan Guo | 2019 | China | 46 | 46 | 27.12±1.25 | 26.34±2.89 | Ovulation-inducing Western medicine+Filiform needle+Electroacupuncture | Ovulation-inducing Western medicine | Three menstrual cycles | 0 | 0 | FSH, LH, T, Overall efficiency |
| Danjuan He | 2020 | China | 62 | 62 | 23.21±4.58 | 23.35±4.26 | Ovulation-inducing Western medicine+Catgut embededment in acupoint | Ovulation-inducing Western medicine | Three menstrual cycles | 0 | 0 | FSH, LH, T, LH/FSH, Overall efficiency, Adverse reaction |
| Jingliu He | 2023 | China | 52 | 52 | 29.41±2.31 | 29.68±2.25 | Ovulation-inducing Western medicine+Filiform needle | Ovulation-inducing Western medicine | Three menstrual cycles | 0 | 0 | FSH, E2, LH, Overall efficiency, Adverse reaction |
| Yuan He | 2020 | China | 35 | 35 | 31.97±3.84 | 32.05±3.63 | Ovulation-inducing Western medicine+Electroacupuncture+Warming needle moxibustion | Ovulation-inducing Western medicine | One menstrual cycle | 0 | 0 | LH, FSH, LH/FSH, T, Overall efficiency |
| Ning Li | 2016 | China | 14 | 14 | N/A | N/A | Ovulation-inducing Western medicine+Filiform needle | Ovulation-inducing Western medicine | Six menstrual cycles | 0 | 0 | LH, FSH, LH/FSH, T |
| Aiqin Huang | 2021 | China | 30 | 30 | 30.54±6.62 | 30.89±6.77 | Ovulation-inducing Western medicine+Filiform needle | Ovulation-inducing Western medicine | Three menstrual cycles | 0 | 0 | LH, FSH, E2, T, Overall efficiency, Ovulation rate, Pregnancy rate |
| Chengyi Huang | 2024 | China | 30 | 30 | 27.23±4.96 | 26.10±5.87 | Ovulation-inducing Western medicine+Filiform needle+Warming needle moxibustion | Ovulation-inducing Western medicine | Three menstrual cycles | 0 | 0 | T, LH, FSH, LH/FSH, Overall efficiency |
| Weimei Huang | 2022 | China | 54 | 54 | 28.01±3.14 | 26.49±2.78 | Ovulation-inducing Western medicine+Catgut embededment in acupoint | Ovulation-inducing Western medicine | Three menstrual cycles | 0 | 0 | T, LH, FSH, LH/FSH, Adverse reaction, Pregnancy rate |
| Xingyu Shan | 2022 | China | 30 | 30 | 29.40±4.25 | 29.50±4.11 | Ovulation-inducing Western medicine+Warming needle moxibustion | Ovulation-inducing Western medicine | Six menstrual cycles | 0 | 0 | LH, FSH, T, E2, Overall efficiency, Pregnancy rate |
| Kunxiang Li | 2019 | China | 50 | 50 | 27.78±5.96 | 28.03±5.82 | Ovulation-inducing Western medicine+Warming needle moxibustion | Ovulation-inducing Western medicine | Three menstrual cycles | 0 | 0 | LH, FSH, E2, Overall efficiency |
| Jing Li | 2009 | China | 23 | 20 | 32.63±4.26 | 32.48±4.45 | Ovulation-inducing Western medicine+Electroacupuncture | Ovulation-inducing Western medicine | One menstrual cycle | 0 | 3 | FSH, LH, E2 |
| Li Li | 2014 | China | 53 | 51 | 27.1±2.5 | 25.2±1.8 | Ovulation-inducing Western medicine+Filiform needle | Ovulation-inducing Western medicine | Six menstrual cycles | 0 | 2 | FSH, LH, LH/FSH, T, Adverse reaction, Ovulation rate Pregnancy rate |
| Saisai Li | 2015 | China | 75 | 75 | 25.1±2.3 | 24.1±2.2 | Ovulation-inducing Western medicine+Filiform needle | Ovulation-inducing Western medicine | Six menstrual cycles | 0 | 0 | LH, LH/FSH, T |
| Xiuyang Li | 2021 | China | 20 | 20 | 28.2±3.8 | 28.1±2.6 | Ovulation-inducing Western medicine+Filiform needle | Ovulation-inducing Western medicine | Three menstrual cycles | 0 | 0 | FSH, LH, E2, T |
| Yonghong Li | 2022 | China | 62 | 62 | 28.05±3.15 | 27.64±3.42 | Ovulation-inducing Western medicine+Filiform needle | Ovulation-inducing Western medicine | Three menstrual cycles | 0 | 0 | FSH, LH, LH/FSH, T, E2, Overall efficiency |
| Jiao Chen | 2024 | China | 58 | 58 | N/A | N/A | Ovulation-inducing Western medicine+Filiform needle+Electroacupuncture | Ovulation-inducing Western medicine | Three menstrual cycles | 0 | 0 | LH, FSH, LH/FSH, E2, T, Ovulation rate, Pregnancy rate |
| Yuane Liu | 2018 | China | 138 | 140 | 30±7 | 31.2±8.1 | Ovulation-inducing Western medicine+Filiform needle | Ovulation-inducing Western medicine | Two menstrual cycles | 2 | 0 | FSH, LH, E2, T, LH/FSH, Ovulation rate, Pregnancy rate |
| Xianwei Lv | 2016 | China | 100 | 100 | 32.00±1.89 | 33.00±1.76 | Ovulation-inducing Western medicine+Filiform needle+Warming needle moxibustion+Electroacupuncture | Ovulation-inducing Western medicine | One menstrual cycle | 0 | 0 | FSH, LH, E2, T, LH/FSH, Overall efficiency |
| Hong Ma | 2016 | China | 30 | 30 | 23±2 | 25±4 | Ovulation-inducing Western medicine+Filiform needle | Ovulation-inducing Western medicine | Three menstrual cycles | 0 | 0 | T, Adverse reaction, Ovulation rate |
| Yujuan Men | 2023 | China | 30 | 30 | 29.4±1.8 | 29.5±1.7 | Ovulation-inducing Western medicine+Filiform needle | Ovulation-inducing Western medicine | Three menstrual cycles | 0 | 0 | FSH, LH, T, LH/FSH, Overall efficiency |
| Xuexia Jiang | 2014 | China | 50 | 50 | 25.23±1.45 | 26.12±1.02 | Ovulation-inducing Western medicine+Catgut embededment in acupoint | Ovulation-inducing Western medicine | Three menstrual cycles | 0 | 0 | FSH, LH, LH/FSH, E2, T |
| Xiaoyan Peng | 2020 | China | 30 | 30 | 28.42±1.32 | 28.32±1.35 | Ovulation-inducing Western medicine+Filiform needle | Ovulation-inducing Western medicine | Three menstrual cycles | 0 | 0 | FSH, LH, T, E2 |
| Shuting Jiang | 2021 | China | 53 | 53 | 29.53±3.48 | 29.93±3.56 | Ovulation-inducing Western medicine+Electroacupuncture+Warming needle moxibustion | Ovulation-inducing Western medicine | Three menstrual cycles | 0 | 0 | FSH, LH, T, Overall efficiency, Adverse reaction |
| Chunmei Quan | 2021 | China | 30 | 30 | 23.80±6.98 | 22.97±6.21 | Ovulation-inducing Western medicine+Filiform needle | Ovulation-inducing Western medicine | Three menstrual cycles | 0 | 0 | LH, FSH, LH/FSH, T, Overall efficiency |
| Yingdan Ruan | 2018 | China | 29 | 28 | 28±4 | 27±5 | Ovulation-inducing Western medicine+Filiform needle+Moxibustion | Ovulation-inducing Western medicine | Three menstrual cycles | 0 | 1 | E2, Ovulation rate, Pregnancy rate |
| Liangying Tang | 2015 | China | 30 | 30 | 23.4±12.4 | 24.2±13.1 | Ovulation-inducing Western medicine+Catgut embededment in acupoint | Ovulation-inducing Western medicine | Three menstrual cycles | 0 | 0 | LH, FSH, LH/FSH, E2, T |
| Chongyang Wang | 2015 | China | 40 | 40 | 28.1±2.8 | 28.9±3.2 | Ovulation-inducing Western medicine+Filiform needle | Ovulation-inducing Western medicine | Four menstrual cycles | 0 | 0 | FSH, LH, T, E2 |
| Fan Wang | 2024 | China | 41 | 41 | 28.45±2.17 | 28.41±2.13 | Ovulation-inducing Western medicine+Filiform needle | Ovulation-inducing Western medicine | Three menstrual cycles | 0 | 0 | E2, LH, FSH, T |
| Juyan Dun | 2018 | China | 30 | 30 | 16.9±1.58 | 17±1.82 | Ovulation-inducing Western medicine+Filiform needle | Ovulation-inducing Western medicine | Three menstrual cycles | 0 | 0 | LH, FSH, T, LH/FSH |
| Pei Ji | 2013 | China | 40 | 40 | 23.3±3.2 | 29.5±4.9 | Ovulation-inducing Western medicine+Filiform needle | Ovulation-inducing Western medicine | Three menstrual cycles | 0 | 0 | T, E2, Overall efficiency, Ovulation rate |
| Jiayuan Wang | 2020 | China | 30 | 30 | 26.43±3.52 | 26.23±3.48 | Ovulation-inducing Western medicine+Filiform needle+Electroacupuncture | Ovulation-inducing Western medicine | Three menstrual cycles | 0 | 0 | LH, FSH, E2, T, Pregnancy rate |
| Jie Wang | 2019 | China | 65 | 65 | 28.61±2.75 | 28.92±2.84 | Ovulation-inducing Western medicine+Acupoint injection | Ovulation-inducing Western medicine | Three menstrual cycles | 0 | 0 | FSH, LH, T, Pregnancy rate |
| Jingxiao Wang | 2022 | China | 60 | 60 | 26.03±6.76 | 27.24±5.21 | Ovulation-inducing Western medicine+Filiform needle | Ovulation-inducing Western medicine | Six menstrual cycles | 0 | 0 | FSH, LH, T, Overall efficiency |
| Xueping Lv | 2007 | China | 30 | 30 | N/A | N/A | Ovulation-inducing Western medicine+Filiform needle+Electroacupuncture | Ovulation-inducing Western medicine | Three menstrual cycles | 0 | 0 | E2, Overall efficiency |
| Yi Wang | 2015 | China | 35 | 35 | N/A | N/A | Ovulation-inducing Western medicine+Moxibustion | Ovulation-inducing Western medicine | Three menstrual cycles | 0 | 0 | FSH, LH, LH/FSH, T |
| Zhenzhi Wei | 2022 | China | 60 | 50 | 29.32±2.90 | 30.01±2.45 | Ovulation-inducing Western medicine+Warming needle moxibustion | Ovulation-inducing Western medicine | One menstrual cycle | 0 | 10 | LH, FSH, T, E2, P, Pregnancy rate |
| Lina Wen | 2023 | China | 42 | 42 | 22.18±1.16 | 21.75±1.09 | Ovulation-inducing Western medicine+Filiform needle | Ovulation-inducing Western medicine | Six menstrual cycles | 0 | 0 | T, E2, LH, FSH, Adverse reaction, Ovulation rate, Pregnancy rate |
| Dan Wu | 2020 | China | 56 | 56 | 28.64±3.73 | 29.43±2.97 | Ovulation-inducing Western medicine+Filiform needle | Ovulation-inducing Western medicine | Three menstrual cycles | 0 | 0 | LH, FSH, T, LH/FSH, Adverse reaction |
| Jiaman Wu | 2021 | China | 50 | 50 | 31±3 | 31±3 | Ovulation-inducing Western medicine+Filiform needle | Ovulation-inducing Western medicine | Three menstrual cycles | 0 | 0 | E2, P, LH, FSH, P, T, Overall efficiency, Pregnancy rate |
| Xiaoming Wu | 2018 | China | 41 | 41 | 29.8±8.1 | 29.4±8.5 | Ovulation-inducing Western medicine+Filiform needle | Ovulation-inducing Western medicine | One menstrual cycle | 0 | 0 | FSH, E2, LH, T, Overall efficiency |
| Shaofang Xiao | 2014 | China | 36 | 36 | 22.36±3.22 | 24.55±3.64 | Ovulation-inducing Western medicine+Catgut embededment in acupoint | Ovulation-inducing Western medicine | Three menstrual cycles | 0 | 0 | LH, FSH, LH/FSH, T, E2, Overall efficiency |
| Yang Xiao | 2021 | China | 62 | 62 | 31±6 | 31±6 | Ovulation-inducing Western medicine+Fire needle | Ovulation-inducing Western medicine | Three menstrual cycles | 0 | 0 | FSH, E2, P, Overall efficiency, Adverse reaction |
| Kai Xie | 2019 | China | 50 | 50 | 25.34±6.16 | 25.32±6.15 | Ovulation-inducing Western medicine+Warming needle moxibustion | Ovulation-inducing Western medicine | Three menstrual cycles | 0 | 0 | FSH, LH, E2, T, Overall efficiency, Pregnancy rate, Ovulation rate |
| Xiaoxue Zhang | 2022 | China | 60 | 60 | 28.52±2.64 | 29.26±3.27 | Ovulation-inducing Western medicine+Warming needle moxibustion | Ovulation-inducing Western medicine | Three menstrual cycles | 0 | 0 | LH, T, Overall efficiency |
| Liwei Xing | 2022 | China | 36 | 36 | 34.85±5.60 | 34.24±5.67 | Ovulation-inducing Western medicine+Filiform needle | Ovulation-inducing Western medicine | Three menstrual cycles | 0 | 0 | FSH, LH, E2, T |
| Caixia Xu | 2020 | China | 41 | 41 | 27.73±5.29 | 28.16±4.39 | Ovulation-inducing Western medicine+Filiform needle+Warming needle moxibustion | Ovulation-inducing Western medicine | Nine menstrual cycles | 0 | 0 | LH, FSH, T, E2, P, Pregnancy rate |
| Xia qiu | 2019 | China | 38 | 37 | 29.07±3.27 | 28.84±3.18 | Ovulation-inducing Western medicine+Filiform needle+Warming needle moxibustion | Ovulation-inducing Western medicine | Three menstrual cycles | 0 | 1 | P, T, E2, LH, FSH, Ovulation rate, Pregnancy rate |
| Zhenghua Xu | 2015 | China | 38 | 38 | 27.51±3.15 | 27.55±3.16 | Ovulation-inducing Western medicine+Filiform needle | Ovulation-inducing Western medicine | Four menstrual cycles | 0 | 0 | FSH, LH, E2, T, LH/FSH, Overall efficiency, Ovulation rate, Pregnancy rate |
| Juan Xu | 2018 | China | 30 | 30 | 25.7±4.0 | 25.8±4.2 | Ovulation-inducing Western medicine+Filiform needle | Ovulation-inducing Western medicine | Two menstrual cycles | 0 | 0 | FSH, LH, E2, T, Pregnancy rate, Ovulation rate |
| Lifeng Xue | 2022 | China | 40 | 40 | 29.45±6.68 | 27.53±3.81 | Ovulation-inducing Western medicine+Filiform needle+Warming needle moxibustion | Ovulation-inducing Western medicine | Three menstrual cycles | 0 | 0 | T, LH, FSH, Overall efficiency, Ovulation rate, Pregnancy rate |
| Tong Li | 2020 | China | 31 | 30 | N/A | N/A | Ovulation-inducing Western medicine+Filiform needle | Ovulation-inducing Western medicine | Three menstrual cycles | 0 | 1 | LH, FSH, LH/FSH, T, Overall efficiency |
| Dajing Yan | 2018 | China | 45 | 45 | 28.56±7.43 | 28.67±8.45 | Ovulation-inducing Western medicine+Catgut embededment in acupoint | Ovulation-inducing Western medicine | N/A | 0 | 0 | LH, FSH, T, E2, P, Adverse reaction, Pregnancy rate |
| Huili Yan | 2023 | China | 30 | 30 | 27.87±5.23 | 27.62±5.35 | Ovulation-inducing Western medicine+Catgut embededment in acupoint | Ovulation-inducing Western medicine | Three menstrual cycles | 0 | 0 | FSH, LH, T, Overall efficiency |
| Baozhi Yang | 2015 | China | 102 | 98 | 31.24±4.72 | 30.99±4.99 | Ovulation-inducing Western medicine+Filiform needle+Electroacupuncture | Ovulation-inducing Western medicine | One menstrual cycle | 0 | 4 | LH, E2, P, Overall efficiency, Pregnancy rate |
| Cheng Yang | 2021 | China | 39 | 39 | 28.61±1.20 | 28.51±1.18 | Ovulation-inducing Western medicine+Filiform needle | Ovulation-inducing Western medicine | Three menstrual cycles | 0 | 0 | LH, FSH, T, E2, Adverse reaction |
| Xueshu Zhang | 2016 | China | 60 | 60 | 28.01±2.31 | 29.45±3.12 | Ovulation-inducing Western medicine+Catgut embededment in acupoint | Ovulation-inducing Western medicine | Three menstrual cycles | 0 | 0 | FSH, LH, E2, T, P, Ovulation rate, Pregnancy rate |
| Ting Yang | 2023 | China | 62 | 59 | 27.90±3.15 | 28.33±4.27 | Ovulation-inducing Western medicine+Warming needle moxibustion | Ovulation-inducing Western medicine | Three menstrual cycles | 0 | 3 | FSH, T, LH, P |
| Xiang Yang | 2018 | China | 46 | 45 | 28.3±4.2 | 28.3±4.2 | Ovulation-inducing Western medicine+Filiform needle | Ovulation-inducing Western medicine | N/A | 0 | 1 | T, LH/FSH, LH, FSH |
| Yamei Yang | 2016 | China | 39 | 39 | 26.9±3.2 | 25.8±2.7 | Ovulation-inducing Western medicine+Catgut embededment in acupoint | Ovulation-inducing Western medicine | Three menstrual cycles | 0 | 0 | FSH, LH, T |
| Ying Xu | 2024 | China | 74 | 74 | 35.62±4.68 | 34.98±5.01 | Ovulation-inducing Western medicine+Catgut embededment in acupoint | Ovulation-inducing Western medicine | Three menstrual cycles | 0 | 0 | FSH, LH, T, Overall efficiency |
| Yongyan Yang | 2021 | China | 58 | 57 | 28.13±5.34 | 28.14±5.29 | Ovulation-inducing Western medicine+Filiform needle | Ovulation-inducing Western medicine | Three menstrual cycles | 0 | 1 | FSH, LH, E2, T, Overall efficiency, Pregnancy rate, Ovulation rate |
| Guochao Yin | 2021 | China | 53 | 53 | 32.75±6.18 | 33.45±6.29 | Ovulation-inducing Western medicine+Filiform needle | Ovulation-inducing Western medicine | Three menstrual cycles | 0 | 0 | LH, FSH, LH/FSH, Overall efficiency |
| Yan Yin | 2017 | China | 56 | 54 | 28.2±4.1 | 28.4±3.09 | Ovulation-inducing Western medicine+Catgut embededment in acupoint | Ovulation-inducing Western medicine | Three menstrual cycles | 0 | 2 | FSH, LH, T, LH/FSH, Overall efficiency, Ovulation rate, Pregnancy rate |
| Dongwen Cui | 2022 | China | 28 | 28 | 26.53±3.11 | 26.25±3.01 | Ovulation-inducing Western medicine+Filiform needle | Ovulation-inducing Western medicine | Three menstrual cycles | 0 | 0 | LH, FSH, E2, P, Overall efficiency, Ovulation rate |
| Liqing Yu | 2018 | China | 40 | 40 | 30±4 | 29±5 | Ovulation-inducing Western medicine+Filiform needle+Electroacupuncture | Ovulation-inducing Western medicine | Three menstrual cycles | 0 | 0 | E2, P, Overall efficiency, Ovulation rate, Pregnancy rate |
| Wenhui Wang | 2021 | China | 30 | 29 | 28.40±5.01 | 28.52±5.55 | Ovulation-inducing Western medicine+Filiform needle+thunder fire moxibustion | Ovulation-inducing Western medicine | Three menstrual cycles | 0 | 1 | FSH, LH, T, Overall efficiency |
| Jin Lu | 2014 | China | 20 | 12 | 25.64±3.95 | 24.75±3.87 | Ovulation-inducing Western medicine+Catgut embededment in acupoint | Ovulation-inducing Western medicine | Three menstrual cycles | 0 | 8 | E2, T, FSH, LH, Overall efficiency |
| Tiejun Ye | 2020 | China | 35 | 35 | 26.3±3.9 | 27.1±3.2 | Ovulation-inducing Western medicine+Moxibustion | Ovulation-inducing Western medicine | Six menstrual cycles | 0 | 0 | T, LH, E2 |
| Lixia Zhang | 2020 | China | 47 | 44 | 27±3 | 26±3 | Ovulation-inducing Western medicine+Catgut embededment in acupoint | Ovulation-inducing Western medicine | Three menstrual cycles | 0 | 3 | FSH, LH, E2, T, Overall efficiency |
| Yaqin Zhang | 2017 | China | 64 | 64 | 28.3±4.2 | 27.9±4.3 | Ovulation-inducing Western medicine+Filiform needle | Ovulation-inducing Western medicine | Three menstrual cycles | 0 | 0 | LH, LH/FSH, T, Overall efficiency |
| Yingxin Zhang | 2022 | China | 30 | 30 | 28.06±5.69 | 27.80±6.14 | Ovulation-inducing Western medicine+Catgut embededment in acupoint | Ovulation-inducing Western medicine | Three menstrual cycles | 0 | 0 | T, E2, P, FSH, LH, Overall efficiency |
| Zeli Zhang | 2016 | China | 50 | 50 | 26.3±4.4 | 27.2±4.1 | Ovulation-inducing Western medicine+Filiform needle | Ovulation-inducing Western medicine | Six menstrual cycles | 0 | 0 | FSH, LH, LH/FSH, T |
| Yunxia Gu | 2018 | China | 62 | 62 | 25.84±4.13 | 26.11±3.82 | Ovulation-inducing Western medicine+Filiform needle | Ovulation-inducing Western medicine | Six menstrual cycles | 0 | 0 | E2, FSH, LH, T, P, Overall efficiency |
| Yuting Chen | 2017 | China | 30 | 30 | 29±6 | 30±6 | Ovulation-inducing Western medicine+Filiform needle+Electroacupuncture | Ovulation-inducing Western medicine | Three menstrual cycles | 0 | 0 | E2, FSH, LH, T, Ovulation rate, Pregnancy rate |
| Ting Zheng | 2024 | China | 40 | 40 | 28.9±2.1 | 28.8±2.2 | Ovulation-inducing Western medicine+Filiform needle | Ovulation-inducing Western medicine | Three menstrual cycles | 0 | 0 | FSH, LH, T, Overall efficiency, Pregnancy rate |
| Yongxia Zheng | 2022 | China | 50 | 50 | 28.62±4.17 | 28.49±3.69 | Ovulation-inducing Western medicine+Warming needle moxibustion | Ovulation-inducing Western medicine | Three menstrual cycles | 0 | 0 | LH, FSH, E2, Overall efficiency, Pregnancy rate |
| Chunhua Zhong | 2023 | China | 31 | 31 | 30.31±2.49 | 30.44±3.19 | Ovulation-inducing Western medicine+Filiform needle+thunder fire moxibustion | Ovulation-inducing Western medicine | Three menstrual cycles | 0 | 0 | LH, T, Overall efficiency |
| Hao Zhong | 2019 | China | 30 | 30 | 26.77±4.797 | 26.57±5.173 | Ovulation-inducing Western medicine+Filiform needle+Moxibustion | Ovulation-inducing Western medicine | Three menstrual cycles | 0 | 0 | FSH, LH, T, Overall efficiency |
| Jinyan Zhong | 2016 | China | 30 | 30 | N/A | N/A | Ovulation-inducing Western medicine+Filiform needle+Electroacupuncture | Ovulation-inducing Western medicine | Three menstrual cycles | 0 | 0 | LH, LH/FSH, T |
| Xiaoyan Zhu | 2015 | China | 42 | 40 | 28.86±4.31 | 29.02±3.48 | Ovulation-inducing Western medicine+Filiform needle+Moxibustion | Ovulation-inducing Western medicine | Three menstrual cycles | 0 | 2 | FSH, LH, E2, P, T, Ovulation rate, Pregnancy rate |
| Haina Zhuang | 2024 | China | 36 | 36 | 29±4 | 29±3 | Ovulation-inducing Western medicine+Filiform needle+Warming needle moxibustion+Electroacupuncture | Ovulation-inducing Western medicine | Three menstrual cycles | 0 | 0 | FSH, LH, E2, T, Ovulation rate, Pregnancy rate |

**Table S3** List of Abbreviations and Intervention Descriptions

| **Abbreviation** | **Full Name** | **Description** |
| --- | --- | --- |
| fn | Filiform needle | The most common form of acupuncture, using thin, solid metallic needles inserted into acupuncture points to regulate qi and blood flow. |
| e | Electroacupuncture | A form of acupuncture in which a small electric current is passed between pairs of acupuncture needles to provide continuous stimulation of acupoints. |
| wnm | Warming needle moxibustion | A combined technique in which moxa (dried mugwort) is attached to the handle of an acupuncture needle and burned, providing simultaneous needling and heat stimulation. |
| ceia | Catgut embededment in acupoint | A technique in which absorbable suture material (catgut) is implanted into acupuncture points, providing prolonged stimulation over days to weeks as the material is absorbed. |
| m | Moxibustion | A traditional Chinese medicine technique involving burning dried mugwort (Artemisia argyi) at or near acupuncture points. Applied as moxa sticks, cones, or boxes. |
| / | Acupoint injection | A technique in which small amounts of liquid substances (such as herbal extracts, vitamins, or medications) are injected into acupuncture points, combining the therapeutic effects of both needling and the pharmacological properties of the injected substance. |
| / | Fire needle | A technique in which a specially designed needle is heated to red-hot in a flame and then rapidly inserted into acupuncture points, combining needle and thermal stimulation. |
| ea | Ear acupuncture | Needling of specific points on the external ear that correspond to body regions or organs, used as a standalone or adjunctive therapy. |
| tfm | Thunder fire moxibustion | A specialised moxa stick containing additional medicinal herbs (e.g., cloves, sandalwood, realgar) that is ignited and held close to acupoints for intense thermal and pharmacological stimulation. |
| / | Western medicine | In this study, the control group received conventional ovulation-inducing pharmacotherapy |

(The moxibustion techniques used in this study include direct moxibustion, stick moxibustion and box moxibustion, which are collectively referred to as moxibustion.)

**The following documents(1-92) are all non-English publications and all come from China.**

1.	Wang JX, Tan L, Zhang TT. Clinical efficacy of kidney-tonifying, phlegm-resolving, and stasis-removing acupuncture as adjuvant therapy for polycystic ovary syndrome. Journal of North Sichuan Medical College. 2022;37(4):505-8,12.

2.	Yang BZ, Cui W, Li J. Effect of electroacupuncture on oocyte quality and pregnancy outcomes in patients with polycystic ovary syndrome. Acupuncture Research. 2015;40(2):151-6.

3.	Cui W, Li J, Sun W, Wen J. Effect of electroacupuncture on oocyte quality and pregnancy in IVF-ET for patients with polycystic ovary syndrome. Chinese Acupuncture & Moxibustion. 2011;31(8):687-91.

4.	Chen J, Cui W, Li J, Sun W. Study on the effect of electroacupuncture intervention on IVF-ET in patients with polycystic ovary syndrome. Maternal and Child Health Care of China. 2009;24(30):4262-4.

5.	Yu LQ, Cao LY, Xie J, Shi Y. Clinical study on electroacupuncture combined with clomiphene for ovulation induction in polycystic ovary syndrome. Chinese Acupuncture & Moxibustion. 2018;38(03):263-8.

6.	He Y, Huang LJ. Clinical observation of electroacupuncture combined with warm needle moxibustion for polycystic ovary syndrome: 35 cases. Massage and Rehabilitation Medicine 2020;11(05):45-6.

7.	Zhong JY, Lu Q, Sun W. Electroacupuncture combined with Diane-35 for polycystic ovary syndrome: 30 cases. Guangming Journal of Chinese Medicine. 2016;31(08):1139-41.

8.	Li J, Cui W, Sun W. Effect of electroacupuncture on IVF-ET pregnancy outcomes in PCOS patients with kidney deficiency. Chinese Journal of Human Sexuality 2009;18(07):28-30.

9.	Li T, Xue L. Therapeutic effect of acupuncture regulating spleen-stomach function on polycystic ovary syndrome. Nei Mongol Journal of Traditional Chinese Medicine 2020;39(2):125-6.

10.	Yang YY. Effect of Ren-Du channel-regulating acupuncture on pregnancy and hormone levels in infertile patients with polycystic ovary syndrome. Modern Medicine and Health Research 2021;5(14):32-5.

11.	Wu JM, Zhuo YY, Tang M, Hu S, Ye YY, Ning Y. Effect of Ren-Du channel-regulating acupuncture on pregnancy outcomes after IVF-ET in infertile PCOS patients. Shanghai Journal of Acupuncture and Moxibustion. 2021;40(5):565-70.

12.	Li YH, Ke Y, Liu YJ, Feng T, Li YC, Li H, et al. Efficacy of Ren-Du channel-regulating acupuncture combined with letrozole for kidney-yang deficiency PCOS infertility and effects on lipid metabolism and sex hormones. Journal of Liaoning University of Traditional Chinese Medicine. 2022;24(7):171-5.

13.	Wang F. Clinical observation of Ren-Du channel-regulating acupuncture combined with Tongyuan acupuncture for PCOS infertility. Journal of Practical Traditional Chinese Medicine. 2024;40(2):261-3.

14.	Liu Y, Cao Y, Wang W, Dai YN. Efficacy of Tung's acupuncture for obese PCOS and its effect on ovarian reserve. World Journal of Integrated Traditional and Western Medicine. 2022;17(7):1431-5.

15.	Wang Y, Xu JH, Hu ZH, Wang SS, Xiong ZM, Bai ZJ, et al. Clinical observation on Governor Vessel moxibustion for spleen-kidney yang deficiency PCOS. Shanghai Journal of Acupuncture and Moxibustion. 2015(1):35-7.

16.	Shan XY, Lai ST. Effect of Governor Vessel warm needle moxibustion as adjuvant therapy for kidney-yang deficiency PCOS infertility. Practical Clinical Journal of Integrated Traditional Chinese and Western Medicine 2022;22(10):105-7.

17.	Xu CX, Mu YY. Efficacy of Governor Vessel warm needle moxibustion combined with clomiphene for PCOS infertility. China Journal of Traditional Chinese Medicine and Pharmacy. 2020;35(4):2153-6.

18.	Wang JY, Ma Y, Du WN, Wang BC, Wu LQ. Clinical study on metformin combined with acupuncture for glycolipid metabolism and adipokines in obese PCOS. China Modern Medicine. 2020;27(27):69-72.

19.	Huang CY, Wang CY. Clinical study on metformin combined with staged acupuncture for obese PCOS. China Prescription Drug. 2024;22(4):162-8.

20.	Ma H, Quan XH, Chen XH, Dong Y. Flying needle acupuncture combined with clomiphene for ovulation disorder in PCOS: A randomized controlled trial. Chinese Acupuncture & Moxibustion. 2016;36(11):1161-5.

21.	Zhai ZY. Efficacy of metformin combined with acupuncture for obese PCOS infertility. International Medicine and Health Guidance News. 2017;23(15):2403-5.

22.	Zhuang HN, Xu JL, Xing LW, He Z, Mei R, Yang ZG, et al. Effect of staged acupuncture combined with medication on ovulation and pregnancy rates in PCOS. Shanghai Journal of Acupuncture and Moxibustion. 2024;43(10):1056-62.

23.	Dun JY, Tian LY, Wang F. Effect of abdominal acupuncture combined with metformin on insulin resistance in adolescent PCOS with phlegm-dampness obstruction. Clinical Journal of Traditional Chinese Medicine. 2018;30(7):1236-9.

24.	Li N, Gong J, Hua C. Clinical study of abdominal acupuncture combined with metformin for obese PCOS. Research of Integrated Traditional Chinese and Western Medicine. 2016;8(1):11-3.

25.	Ji P, Xie B, Wang H. Clinical study of abdominal acupuncture combined with clomiphene for ovulation induction in PCOS. Guiding Journal of Traditional Chinese Medicine and Pharmacy. 2013(7):66-7,8.

26.	Yin Y, Zhang YC, Gao YP, Zhao PC, Guo GR. Modified catgut embedding combined with letrozole for ovulation induction and pregnancy in infertile PCOS patients: 56 cases. Jiangsu Journal of Traditional Chinese Medicine. 2017;49(7):57-9.

27.	Xiao Y, Wang YP. Effect of fire needle on sex hormones, ovulation, and endometrial receptivity in infertile PCOS patients. Shanghai Journal of Acupuncture and Moxibustion. 2021;40(9):1106-11.

28.	Xing LW, Liu F, Dong QZ, Wu KN, He Z, Sun YH, et al. Efficacy of "Chong Meridian as Sea of Blood"-based staged acupuncture for PCOS frozen embryo transfer. Proceedings of the 13th National Forum for Doctoral Candidates in Traditional Chinese Medicine; Shenyang2022. p. 2755-8.

29.	Chen J, Fan HY, Zeng JZ, Liu Y, Hu JQ, Zhou MQ, et al. .Clinical efficacy of "Ren Meridian Governing Fetus"-based electroacupuncture cycle therapy combined with clomiphene citrate for PCOS. China Journal of Traditional Chinese Medicine and Pharmacy 2024;39(1):496-9.

30.	Zhong CH, Hu SY, Lin QY. Effect analysis of constitution-based acupuncture for PCOS. China Foreign Medical Treatment. 2023;42(35):186-9.

31.	Ye TJ, Cheng HX. Efficacy of moxibustion combined with herbs for PCOS infertility and effects on serum inflammatory factors. Journal of Acupuncture and Tuina Science 2020;18(4):269-75.

32.	Guo ZS. Oral cyproterone acetate/ethinylestradiol tablets combined with acupuncture for PCOS infertility. Practical Clinical Journal of Integrated Traditional Chinese and Western Medicine. 2019;19(11):39-40.

33.	Wu XM. Clinical application of letrozole combined with acupuncture for clomiphene-resistant PCOS. China Medicine and Pharmacy. 2018;8(8):49-51.

34.	Huang AQ. Effect of Lai's Tongyuan acupuncture combined with conventional Western medicine on PCOS infertility. Medical Journal of Chinese People's Health. 2021;33(15):91-3.

35.	Wen LN, Wang M, Zhou YH. Efficacy of Longsha Kaiheshu acupuncture combined with letrozole for PCOS infertility. China Medical Herald 2023;20(30):117-20.

36.	Zheng T, Li D, Li AX, Xiang S, Li D, Liu Y. Efficacy of umbilical acupuncture combined with letrozole for kidney deficiency-phlegm dampness PCOS with infertility. Electronic Journal of Practical Gynecological Endocrinology. 2024;11(13):30-4.

37.	Fu HY, Du HS, Song XG, Ma JX, Lu N. Effect of periumbilical eight-point catgut embedding on IVF-ET outcomes in obese PCOS infertility. Shandong Journal of Traditional Chinese Medicine. 2024;43(1):53-8.

38.	He JL, Li PZ. Efficacy analysis of periumbilical eight-point acupuncture combined with letrozole for obese PCOS. Chronic Pathematology Journal 2023;24(11):1699-701+708.

39.	Yin GC, Yang JW, Chen NH, Zhang YD, Qin GQ, Duan JL. Clinical efficacy of pulse-based acupuncture for obese PCOS infertility and correlation with meridian energy. Journal of Translational Medicine. 2021;10(4):238-41.

40.	Gan XL, Deng WH. Clinical study on Governor-Conception Vessel moxibustion combined with catgut embedding for obese PCOS. Chinese Medicine Modern Distance Education of China. 2022;20(18):98-101.

41.	Zhang XS, Cao WF, Yang H. Randomized controlled trial of Shuixue (EX-LE2) catgut embedding combined with clomiphene for obese PCOS infertility. Journal of Chengdu University of Traditional Chinese Medicine 2016;39(1):67-70.

42.	Chen YT, Li YM, Luo JL, Chen YJ, Zheng SZ. Clinical observation of Tongyuan acupuncture combined with medication for refractory PCOS infertility. Shanghai Journal of Acupuncture and Moxibustion 2017;36(6):692-6.

43.	Lu J, Zhang XS, Zhou X, Zhou HF, Li J, Zhang CR. Microinvasive catgut embedding combined with Diane-35 for obese PCOS: 20 cases. Jiangsu Journal of Traditional Chinese Medicine. 2014(7):55-6.

44.	Guo WX, Li JC, Wang HF. Efficacy evaluation of Governor Vessel warm needle moxibustion for PCOS infertility. World Latest Medicine Information. 2021;21(96):191-2.

45.	Zheng YX, Yu JW, Mai WT. Efficacy of warm needle moxibustion combined with letrozole for kidney deficiency-blood stasis PCOS infertility. Journal of Practical Traditional Chinese Medicine. 2022;38(8):1368-9.

46.	Yang T, Tan JJ, Yang J. Effects of warm needle moxibustion combined with conventional Western medicine on ovarian function, pregnancy, and sex hormones in PCOS infertility. Hebei Journal of Traditional Chinese Medicine. 2023;45(9):1545-8.

47.	Wei ZZ, Zhang DD, Song HF, He XM. Clinical study of warm needle moxibustion combined with clomiphene and progesterone for kidney deficiency PCOS infertility. Journal of Shanxi Health Vocational College. 2022;32(3):70-1.

48.	Xue LF, Miao YX. Efficacy of warm needle moxibustion combined with clomiphene for PCOS infertility and effects on sex hormones and ovulation. New Chinese Medicine. 2022;54(10):164-7.

49.	Jiang ST, Chen SF, Qi CH. Clinical study of warm needle moxibustion combined with clomiphene for polycystic ovary syndrome. Doctor. 2021;6(11):75-7.

50.	Li KX. Clinical value of warm needle moxibustion for kidney deficiency-phlegm dampness PCOS. Practical Clinical Journal of Integrated Traditional Chinese and Western Medicine 2019;19(01):34-6.

51.	Li XY, Hao MJ, Lu N, Wang XC, Ma JX, Fu HY. Effect of catgut embedding on follicular microenvironment and IVF-ET pregnancy outcomes in PCOS with insulin resistance. Chinese Journal of Family Planning & Gynecotokology 2022;14(11):48-52.

52.	Tang LY, Huang RC, Zhang JF. Effect of catgut embedding on sex hormone-related indices in obese PCOS. Clinical Journal of Chinese Medicine. 2015(21):60-2.

53.	Yang YM. Clinical efficacy of catgut embedding combined with Diane-35 on endocrine metabolic abnormalities in PCOS. Journal of Clinical Acupuncture and Moxibustion. 2016;32(6):33-5.

54.	Xiao SF, Xu YY, Zhu LJ, Hu JL. Catgut embedding combined with Diane and metformin for obese PCOS: 36 cases. Jiangxi Journal of Traditional Chinese Medicine 2014(4):53-5.

55.	Zhang LX, Li DP. Efficacy of catgut embedding combined with cyproterone acetate/ethinylestradiol for PCOS. Shanghai Journal of Acupuncture and Moxibustion. 2020;39(3):339-43.

56.	Jiang XX, Meng J. Clinical observation of catgut embedding combined with metformin hydrochloride for insulin resistance in kidney deficiency-phlegm dampness PCOS: 50 cases. Hebei Journal of Traditional Chinese Medicine 2014(2):171-4.

57.	Zhang YX. Clinical efficacy analysis of catgut embedding combined with Diane-35 for polycystic ovary syndrome. Journal of Aerospace Medicine. 2022;33(2):215-8.

58.	Yan HL, Zhou LY, Zhang L, Shen XJ. Clinical observation of catgut embedding for overweight/obese PCOS. Guangming Journal of Chinese Medicine. 2023;38(4):669-72.

59.	Xu Y, Mi HX, Yang Y. Effect of catgut embedding on metabolism and hormone levels in PCOS. Chinese Journal of General Practice 2024;22(4):665-8.

60.	Huang WM, Xia DB, Wang XLL, H.C., Ouyang HL. Clinical study on catgut embedding for polycystic ovary syndrome. Asia-Pacific Traditional Medicine. 2022;18(9):90-5.

61.	He DJ, Liang SR, Huang XT. Clinical study of catgut embedding for obese PCOS. Hubei Journal of Traditional Chinese Medicine 2020;42(02):12-6.

62.	Chen L. Clinical efficacy of catgut embedding for clomiphene-resistant PCOS. China Practical Medicine. 2019;14(32):58-60.

63.	Wang J, Yang T. Effect of acupoint application combined with clomiphene on kidney deficiency-blood stasis PCOS with infertility. Journal of Clinical Medicine in Practice. 2019;23(21):74-8.

64.	Yan DJ, Zhang LL, Zhang B, Liu K, Xue XF, Zhang X, et al. Clinical observation of nutritional therapy combined with catgut embedding for obese PCOS. Clinical Misdiagnosis & Mistherapy 2018;31(12):70-5.

65.	Dai XF. Clinical observation of acupuncture, ear acupressure combined with metformin for obese PCOS infertility. New Chinese Medicine. 2017;49(4):128-31.

66.	Cui DW, Mi JP, Yu ZS. Effect of acupuncture at "New Baliao Points" on ovulation and TCM symptom scores in PCOS infertility. Journal of Hubei University of Chinese Medicine. 2022;24(6):36-9.

67.	Men YJ, Chen PF, Wang RY, Chen YJ, Xia DP, Zhang XF. Clinical observation of acupuncture at Baliao points for kidney-yang deficiency PCOS-induced menstrual disorders. Guangxi Journal of Traditional Chinese Medicine. 2023;46(4):24-7.

68.	Xu J, Zuo Y. Therapeutic effect of acupuncture as adjuvant therapy for infertile patients with polycystic ovary syndrome. Chinese Acupuncture & Moxibustion 2018;38(4):358-61,63.

69.	Xu ZH, Wang YF. Therapeutic effect of acupuncture combined with ovulation induction on phlegm-dampness PCOS infertility. Women's Health Research. 2015(24):233-,6.

70.	Li XY, Li XX, Zhang N, Ren J. Study on the targets of acupuncture combined with Diane-35 for phlegm-dampness PCOS. Modern Journal of Integrated Traditional Chinese and Western Medicine 2021;30(5):463-8,506.

71.	Peng XY. Clinical observation of acupuncture combined with metformin for polycystic ovary infertility. Chinese Community Doctors. 2020;36(26):121-2.

72.	Liu YE, Liao BD. Clinical observation of acupuncture combined with metformin for polycystic ovary infertility. Shanghai Journal of Acupuncture and Moxibustion 2018;37(12):1354-8.

73.	Quan CM, Zhang ZJ, He XL, Cao H, Liu W, Lei MH. Acupuncture combined with metformin for spleen deficiency-phlegm dampness PCOS: 60 cases. Hunan Journal of Traditional Chinese Medicine. 2021;37(11):73-5,118.

74.	Wang CY. Clinical efficacy observation of acupuncture combined with clomiphene for polycystic ovary syndrome. Asia-Pacific Traditional Medicine. 2015;11(11):83-5.

75.	Wang WH, Liu P, Shou Y, Zhang KY, Yuan L, Li BR, et al. Clinical study of acupuncture combined with thunder-fire moxibustion for spleen-kidney deficiency PCOS. China Prescription Drug. 2021;19(9):158-61.

76.	Zhong H, Zhang Z. Clinical efficacy of acupuncture at Baliao points combined with Diane-35 for PCOS-induced menstrual disorders. Journal of Tianjin University of Traditional Chinese Medicine 2019(6):583-7.

77.	Yang C, Yang BX. Study on the improvement of ovarian function by acupuncture in obese PCOS patients. World Latest Medicine Information. 2021;21(54):89-90.

78.	Wu D, Wang XB, Cong HF, Liu SM, Liang Y. Effect of acupuncture on ovarian function in obese PCOS patients. World Chinese Medicine. 2020;15(16):2482-5.

79.	Zhang YQ. Effect of acupuncture on adiponectin, leptin and insulin resistance in obese PCOS patients. Acta Chinese Medicine. 2017;32(11):2259-62.

80.	Li SS. Acupuncture adjuvant to metformin for obese PCOS infertility: 75 cases. Chinese Medicine Modern Distance Education of China 2015(6):78-9.

81.	Zhang ZL. Efficacy of acupuncture combined with metformin for obese PCOS infertility. Primary Medical Forum. 2016;20(33):4670-1.

82.	Yang X. Clinical effect of acupuncture combined with metformin for obese PCOS infertility. Healthmust-Readmagazine. 2018(3):21-2.

83.	Li L, Mo H, Wen B, Zhang J, Li Y, Chen WF, et al. Clinical study of acupuncture combined with metformin for obese PCOS infertility. China Journal of Traditional Chinese Medicine and Pharmacy. 2014;29(7):2115-9.

84.	Gu YX, Li J, Zheng PY. Effect of acupuncture combined with Western medicine on hormone levels and ovarian blood flow in PCOS infertility. Primary Medical Forum 2018;22(25):3588-90.

85.	Zhang XX, Wang HL, Xie CC. Efficacy of acupuncture combined with Western medicine for obese PCOS patients. Journal of Hubei University of Chinese Medicine 2022;24(6):86-8.

86.	Xie K, Wang LL. Efficacy and prognosis analysis of acupuncture for polycystic ovary syndrome. Chinese Journal of General Practice. 2019;17(5):834-6.

87.	Zhu XY, Ji LP, Liu CY, Wu GH. Clinical effect of acupuncture for PCOS with infertility. World Journal of Integrated Traditional and Western Medicine 2015(12):1720-2.

88.	Lyu XW. Clinical effect observation of acupuncture for PCOS with infertility. Nei Mongol Journal of Traditional Chinese Medicine. 2016;35(15):119-20.

89.	Qiu X, Xu MY. Effect of acupuncture on PCOS with infertility. Journal of Clinical Rational Drug Use. 2019;12(1).

90.	Wang C, Wu ZL, Chen SM, Chen ZX, Chen R, Chen XH. Retrospective analysis of cycle-based acupuncture combined with Back-Shu/Front-Mu points for obese PCOS. Journal of Guangzhou University of Traditional Chinese Medicine. 2021;38(11):2428-33.

91.	Si XP, Cong HF, Wang XB. Therapeutic effect of acupuncture combined with herbs on ovulation disorder in PCOS. Journal of Clinical Acupuncture and Moxibustion. 2007;23(8):35-6.

92.	Ruan YD, Yan YT, Wei RH, Shen D. Clinical study of acupuncture combined with herbs for clomiphene-resistant PCOS infertility. Shanghai Journal of Acupuncture and Moxibustion. 2018;37(7):769-72.
