# Supplementary material for: Efficacy of acupuncture therapies for polycystic ovary syndrome: a network meta-analysis
Source: Front Med (Lausanne). 2026 Jul 29;13:1895421. doi: 10.3389/fmed.2026.1895421 (PMC13461588; doi:10.3389/fmed.2026.1895421)
Supplement: Supplementary file 3 [file Table_3.DOCX]

The sensitivity analysis of FSH involves the "fire needle" therapy.

Figure 1	FSH (without fire needle)

Figure 2	FSH

The top three results of the SUCRA probability ranking in Figure 1 show: Fn+e (79.72%) > Fn+wnm+e (70.59%) > Fn+wnm (67%)

The top four results of the SUCRA probability ranking in Figure 2 show: Fire needle (90.99%) > Fn+e (75.29%) > Fn+wnm+e (66.76%) > Fn+wnm (63.16%)

The SUCRA sorting result was not affected.

The sensitivity analysis of AEs involves the "fire needle" therapy.

Figure 3	AEs (without fire needle)
Figure 4	AEs

The top three results of the SUCRA probability ranking in Figure 3 show: Western medicine (96.09%) > Catgut embededment in acupoint (74.28%) > Filiform needle (59.95%)

The top four results of the SUCRA probability ranking in Figure 4 show: Fire needle（91.69%）>Western medicine（83.17%）>Catgut embededment in acupoint（64.08%）>Filiform needle（51.49%）

The SUCRA sorting result was not affected.

The sensitivity analysis of T involves the "CEIA + M" therapy.

Figure 5	T (without CEIA + M)

Figure 6	T

The top three results of the SUCRA probability ranking in Figure 5 show: Fn+m(99.99%) > Electroacupuncture (87.88%) > Fn+wnm (63.35%)

The top four results of the SUCRA probability ranking in Figure 6 show: Fn+m（96.53%）>Ceia+m（85.64%）>Electroacupuncture（82.33%）>Fn+wnm（59.46%）

The SUCRA sorting result was not affected.
