# Supplementary material for: Efficacy of acupuncture therapies for polycystic ovary syndrome: a network meta-analysis
Source: Front Med (Lausanne). 2026 Jul 29;13:1895421. doi: 10.3389/fmed.2026.1895421 (PMC13461588; doi:10.3389/fmed.2026.1895421)
Supplement: Supplementary file 4 [file Table_4.DOCX]

**Total efficacy (TE)**

The SUCRA ranking of TE was as follows: electroacupuncture (99.03%) > FN+TFM (74.77%) > FN+E (64.02%) > FN+WNM (60.77%) > FN+M (59.72%) > CEIA (58.61%) > moxibustion (46.04%) > FN (42.86%) > WNM (40.52%) > FN+WNM+E (27.13%) > fire needle (26.05%) > Western medicine (0.43%) (Figure 4A).

**Follicle-stimulating hormone (FSH)**

The SUCRA ranking for FSH levels was as follows: fire needle (90.99%) > FN+E (75.29%) > FN+WNM+E (66.76%) > FN+WNM (63.16%) > FN (61.45%) > Western medicine (60.63%) > FN+TFM (59.05%) > CEIA (58.91%) > moxibustion (52.50%) > FN+M (47.41%) > WNM (42.88%) > WNM+E (30.94%) > acupoint injection (22.52%) > electroacupuncture (17.31%) > FN+EA (0.13%) (Figure 4B).

**Luteinizing hormone (LH)**

The SUCRA-based ranking for LH reduction was as follows: WNM (86.75%) > WNM+E (72.76%) > FN (69.55%) > FN+WNM+E (68.96%) > FN+M (68.61%) > FN+WNM (58.77%) > FN+TFM (50.38%) > CEIA (47.81%) > moxibustion (47.60%) > FN+E (44.38%) > FN+EA (35.08%) > acupoint injection (31.16%) > Western medicine (10.63%) > electroacupuncture (7.50%) (Figure 4C).

**Testosterone**

The SUCRA ranking for reducing testosterone levels was as follows: FN+M (96.53%) > CEIA+M (85.64%) > electroacupuncture (82.33%) > FN+WNM (59.46%) > CEIA (48.85%) > FN+WNM+E (47.44%) > moxibustion (47.05%) > FN (46.64%) > FN+E (40.44%) > acupoint injection (38.46%) > WNM (26.94%) > (21.07%) > Western medicine (8.87%) (Figure 4D).

**LH/FSH ratio**

The SUCRA-based ranking for reducing LH/FSH ratio levels was as follows: CEIA (77.73%) > WNM+E (72.87%) > FN+WNM+E (66.39%) > FN (61.93%) > FN+E (56.78%) > FN+WNM (47.04%) > CEIA+M (35.81%) > moxibustion (17.82%) > Western medicine (13.58%) (Figure 4E).

**Adverse events (AEs)**

The SUCRA-based ranking for safety (from least safe to safest) was as follows: fire needle (91.69%) > Western medicine (83.17%) > CEIA (64.08%) > FN (51.49%) > FN+E (25.32%) > WNM+E (21.32%) > WNM (12.89%) (Figure 4F).
